# Hsp90 and cochaperones have two genetically distinct roles in regulating eEF2 function

**DOI:** 10.1371/journal.pgen.1011508

**Published:** 2024-12-09

**Authors:** Melody D. Fulton, Danielle J. Yama, Ella Dahl, Jill L. Johnson

**Affiliations:** Department of Biological Sciences, University of Idaho, Moscow, Idaho, United States of America; Universite de Montreal, CANADA

## Abstract

Protein homeostasis relies on the accurate translation and folding of newly synthesized proteins. Eukaryotic elongation factor 2 (eEF2) promotes GTP-dependent translocation of the ribosome during translation. eEF2 folding was recently shown to be dependent on Hsp90 as well as the cochaperones Hgh1, Cns1, and Cpr7. We examined the requirement for Hsp90 and cochaperones more closely and found that Hsp90 and cochaperones have two distinct roles in regulating eEF2 function. Yeast expressing one group of Hsp90 mutations or one group of cochaperone mutations had reduced steady-state levels of eEF2. The growth of Hsp90 mutants that affected eEF2 accumulation was also negatively affected by deletion of the gene encoding Hgh1. Further, mutations in yeast eEF2 that mimic disease-associated mutations in human eEF2 were negatively impacted by loss of Hgh1 and growth of one mutant was partially rescued by overexpression of Hgh1. In contrast, yeast expressing different groups of Hsp90 mutations or a different cochaperone mutation had altered sensitivity to diphtheria toxin, which is dictated by a unique posttranslational modification on eEF2. Our results provide further evidence that Hsp90 contributes to proteostasis not just by assisting protein folding, but also by enabling accurate translation of newly synthesized proteins. In addition, these results provide further evidence that yeast Hsp90 mutants have distinct *in vivo* effects that correlate with defects in subsets of cochaperones.

## Author summary

The Hsp90 molecular chaperone and its associated cochaperones are required for the folding of approximately 20% of the yeast proteome. Using a series of yeast Hsp90 mutants defective at distinct steps in the Hsp90 folding pathway, we analyzed the role for Hsp90 and cochaperones in maturation of eukaryotic elongation factor 2, which is required for ribosome translocation. Our results suggest that Hsp90 and cochaperones have two genetically distinct roles in mediating eEF2 function. The folding of eEF2 is highly dependent on the Hgh1, Cpr7, and Cns1 cochaperones and most sensitive to the Hsp90 mutants that disrupt one step in the pathway. In contrast, sensitivity to diphtheria toxin was affected by deletion of the Sti1 cochaperone and mutations that disrupt other steps in the pathway. Thus, it appears that even for a single client, Hsp90 may use different mechanisms to promote different aspects of client maturation. Our results also provide further evidence that Hsp90 contributes to proteostasis, both by assisting protein folding and by supporting folding of proteins required for accurate translation.

## Introduction

The molecular chaperone Hsp90 is required for maintaining up to 20% of the yeast proteome in an active, folded conformation [1–5]. Hsp90 functions as a dimer and undergoes dramatic nucleotide-induced conformational changes [6]. The most complete model of Hsp90 function is based on the ATP-dependent cycle of interaction with glucocorticoid receptor (GR) [7–10]. Unfolded GR progresses through a loading complex consisting of Hsp70, Hsp90, and Sti1/Hop to a mature complex of GR, Hsp90, and the Sba1/p23 and FKBP52 cochaperones. Once in the mature complex, the GR achieves the ability to bind hormone with high affinity. Cryo-EM structures of Hsp90 and cochaperones in complex with GR demonstrate that each cochaperone makes direct contact with the GR, providing the basis for cochaperone-client selectivity [11–13]. However, evidence suggests GR and/or other clients may use a variety of mechanisms that include different cochaperones capable of targeting clients to Hsp90 [14–18]. Moreover, increasing evidence suggests that clients interact with different subsets of cochaperones, and some cochaperones form functional networks [19,20]. There are about twelve Hsp90 cochaperones in yeast and fifty in humans [6,21]. One of our goals is to identify differences in how clients interact with Hsp90 and cochaperones in order to provide insight into potential mechanisms of selective inhibition of Hsp90 function.

The two isoforms of *Saccharomyces cerevisiae* Hsp90 (encoded by *HSC82* and *HSP82*) are 97% identical. Yeast eEF2 (encoded by *EFT1* and *EFT2)* consists of two isoforms that are 100% identical [22,23]. Both proteins are very abundant, with estimates of ~229,000 polypeptides of Hsc82/Hsp82 per cell and 145,000 polypeptides of Eft1/Eft2 [24]. eEF2 has a critical role in ribosome translocation, and mutations in eEF2 result in reduced translational fidelity, including an increase in frameshift mutations [25–27]. Mutations in human eEF2 result in nervous system abnormalities or developmental defects, including non-specific craniofacial dysmorphisms, abnormal brain morphology, and an autosomal dominant form of spinocerebellar ataxia [28,29]. eEF2 undergoes a unique posttranslational diphthamide modification that is conserved in all eukaryotes and archaebacteria. Diphthamide is a modification of a histidine residue in eEF2 (H699 in yeast), and it is the target of diphtheria toxin (DT) and other bacterial ADP-ribosylating toxins [30]. Diphthamide helps stabilize the anticodon-codon interaction, and it is required for maintenance of the accurate reading frame [26,31,32]. The folding of eEF2 was recently shown to be dependent on two cytosolic chaperones, the TRiC/CCT system and the Hsp90 machine. A novel cochaperone, Hgh1, was shown to directly interact with eEF2 and link it to Hsp90 via the Cns1 cochaperone. In the presence of an Hsp90 inhibitor, or in cells expressing reduced levels of the Hgh1, Cpr7, or Cns1 cochaperones, eEF2 becomes misfolded and/or aggregated [33,34].

We developed a series of yeast Hsp90 mutants, each with a single amino acid alteration in the Hsc82 isoform, that we grouped based on shared *in vivo* phenotypes and their ability to disrupt intermediate steps within the folding pathway. The mutants have varied effects on activity of select clients and sensitivity to Hsp90 inhibitors [35–37]. Many of the most studied Hsp90 mutants that have demonstrated defects in on activity of protein kinases and steroid hormone receptors fall into our ’loading and closing’ category. In contrast, we have not previously identified client defects that are strongest in cells expressing mutants in the ’reopening’ group. Here, we examined the effect of Hsp90 or cochaperone mutation on two distinct aspects of the function of eEF2, a recently identified Hsp90 client. We monitored eEF2 folding, as measured by steady-state levels of soluble eEF2, and susceptibility to diphtheria toxin. Our results suggest that Hsp90 and cochaperones have two genetically distinct roles in mediating eEF2 function. The folding of eEF2 is highly dependent on the Hgh1, Cpr7 and Cns1 cochaperones and most sensitive to the Hsp90 mutants in the reopening group. In contrast, sensitivity to diphtheria toxin is dependent on the Sti1 cochaperone and most sensitive to mutations in the loading and closing groups. These results demonstrate that the role of the Hsp90 machinery may vary as a single client progresses to the functional state.

## Material and methods

### Media, chemicals, and antibodies

Standard yeast genetic methods were employed. Yeast were transformed by lithium acetate methods and were grown in either YPD (1% Bacto yeast extract, 2% peptone, and 2% dextrose) or defined synthetic complete media supplemented with 2% dextrose. Plasmid shuffling was used to swap in plasmids expressing wild-type or mutant His-eEF2, Hsc82 or Cns1. Transformants were cured of *URA3* plasmids using 5-fluoroorotic acid (5-FOA) obtained from Toronto Research Chemicals. Galactose was obtained from Fisher. Raffinose was obtained from Acros Organics (Cat. 19567–1000). Antibodies used: polyclonal rabbit anti-eEF2 (Kerafast, ED7002), monoclonal mouse anti-PGK1 (Invitrogen, 459250), goat anti-rabbit HRP (Invitrogen, 32460), goat anti-mouse HRP (Invitrogen, 62–6520), rabbit polyclonal anti-Tim44 [35].

### Plasmids

Plasmids expressing wild-type *EFT1* on a *URA3* plasmid and *LEU2* plasmids expressing wild-type or mutant His-EFT2 (YCpEFT2_6xHis-LEU2) were obtained from the laboratory of J. Dinman (University of Massachusetts) [28]. The control plasmid for the His-EFT2 LEU2 plasmid was YCplacIII [38]. Plasmids expressing wild-type or mutant Hsc82, Cns1, or Cpr7 have been described previously [35,39,40]. *In vivo* diphthamide biosynthesis was monitored by scoring viability of yeast cells upon conditional expression of a galactose-inducible diphtheria toxin (GAL-DT) plasmid pLMY101 [41]. The plasmid pRS424-*HGH1* was constructed by inserting the coding sequence for Hgh1, along with approximately 1 kb upstream and 300 bp downstream flanking nucleotides into pRS424 [42]. The coding sequence of *HGH1* in the plasmid was sequenced completely.

### Yeast strain construction

Yeast strains are isogenic to W303 and are listed in **S1 Table**. Yeast strains containing individual deletions of *EFT1*, *EFT2*, *HGH1* or *DPH2* (*eft1*::*kan*^*R*^, *eft2*::*kan*^*R*^, *hgh1*::*kan*^*R*^ or *dph2*::*kanr*^*R*^) were obtained from the knockout library collection (Horizon Discovery). These strains were back-crossed five times with JJ762 [43] and deletions were confirmed using PCR. The *eft1eft2/URA3*-*EFT1* strain was obtained by mating the individual *eft1* and *eft2* strains and transforming the *URA3-EFT1* plasmid into the diploid prior to mating and tetrad dissection. Plasmids expressing His-eEF2 were transformed into strain JJ1472 (*eft1eft2*) and grown in the presence of 5-FOA to cure the *URA3-EFT1* plasmid. The *hgh1*::*TRP1* strain was constructed from *hgh1*::*kan*^*R*^ using a linearized marker-swap plasmid [44], then crossed to JJ1472 to obtain *eft1eft2hgh1/URA3-EFT1* (JJ1481).

### Isolation of His-eEF2 complexes and immunoblot analysis

His-eEF2 complexes were isolated as described for isolation of His-Hsc82 complexes (26). Cells were cultured in 50 mL of YPD or selective media overnight and harvested at an OD_600_ of 1.7 to 3.2. Cell pellets were stored at -80°C then thawed on ice before resuspending in ice-cold cell lysis buffer (20 mM Tris pH 7.5, 100 mM KCl, 10 mM MoO_4_, 5 mM MgCl_2_, 5 mM imidazole) prepared with a complete Mini protease inhibitor cocktail tablet (Roche Diagnostics). The cells were disrupted by vortexing with glass beads at 4°C for 30 sec and resting for 1 min, repeating these steps 8 times. Crude lysates were precleared by centrifugation at 4,500 x g for 10 sec at 4°C and cleared at 18,000 x g for 5 min at 4° C. Protein complexes were isolated by incubation rocked with PerfectPro Ni-NTA agarose resin for 1 h at 4°C followed by four washes with lysis buffer (20 mM Tris pH 7.5, 102 mM KCl, 10 mM MoO_4_, 5 mM MgCl_2_, 35 mM imidazole, 0.1% Tween 20). The lysate and resin samples were mixed with 2X sample loading buffer (250 mM Tris pH 6.8, 2% SDS, 20% glycerol, 0.05% bromophenol blue and 4% beta-mercaptoethanol) and boiled at 100°C for 3 min. Protein complexes were separated by gel electrophoresis followed by Coomassie Blue staining and/or immunoblot analysis using indicated antibodies. The level of His-eEF2 bound to resin was quantified by normalization to levels of a protein in the stained gels of approximately 35 kDa that binds resin in cells lacking His-eEF2. This method of normalization was used to focus on the plasmid-borne, soluble His-eEF2. Another reason for using this approach was because we observed reproducibly low Pgk1 levels, our loading control, in strains expressing *cns1-G90D*, and some *hsc82* mutants.

### Growth tests, quantification and statistical analysis

The relative growth of the yeast cells was measured using spotting assays and quantification as described elsewhere [45]. Cells were inoculated into 5 mL of YPD media (or other media as indicated) and incubated in a shaking incubator at 30°C overnight. Growth was examined by spotting 10-fold serial dilutions of normalized yeast cultures on appropriate media, followed by incubation for two days at 30°C or 37°C, or other conditions as indicated. Spotting assays contained respective control lanes on each experimental plate. ImageJ was used to obtain pixel counts of the second dilution series (unless otherwise indicated). Values were normalized to the average of the respective control lanes. For all growth assays, a minimum of 3 biological replicates were used. Statistical significance was evaluated with GraphPad Prism using an ordinary one-way ANOVA or Mixed-effects analysis as indicated (* P ≤ 0.05; ** P ≤ 0.01; *** P ≤ 0.001; **** P ≤ 0.0001). Non-significant values (P ≥ 0.05 not shown). Error bars represent standard errors of the mean.

## Results

### Effect of cochaperone mutation on eEF2 steady-state levels

**Fig 1:**
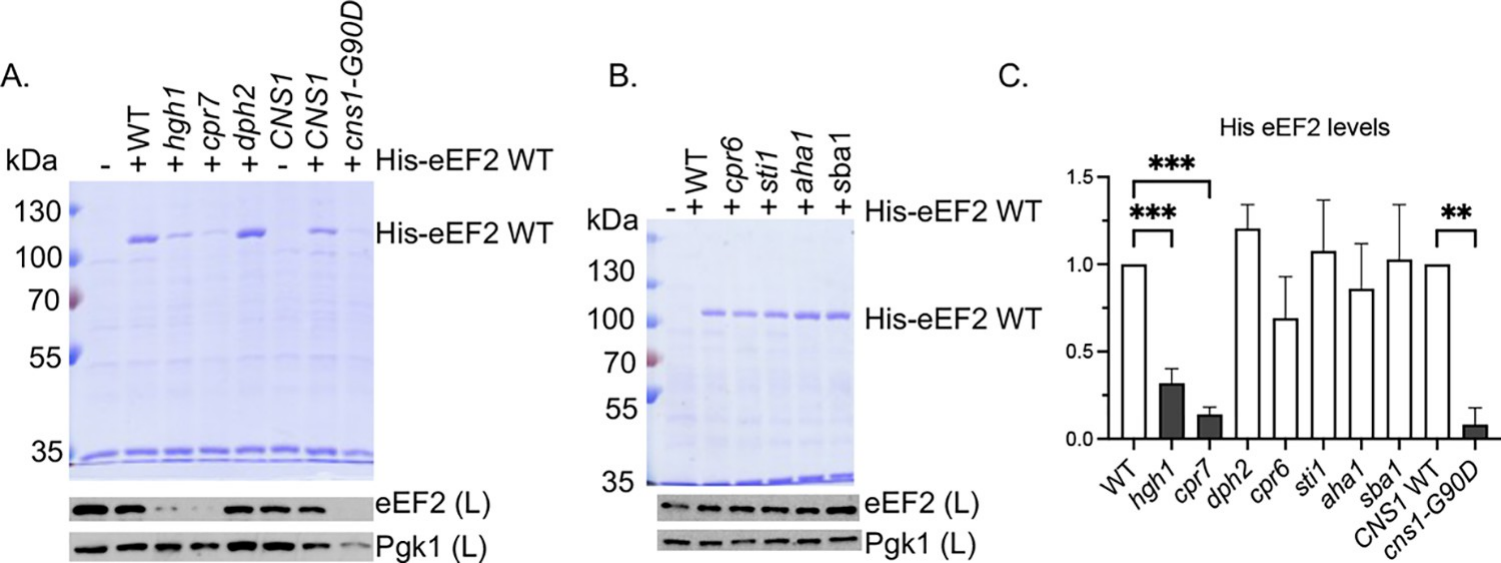
Effect of cochaperone deletion or mutation 6x-His eEF2 levels. His-eEF2 was isolated from the indicated strain **(S1 Table)** using nickel resin. Proteins bound to the nickel resin were separated using SDS-PAGE and visualized using stained gels (top). Levels of eEF2 were also detected using immunoblot analysis of whole cell lysates (below). Lane 1: empty vector (YcPlac111). eEF2 levels in the immunoblots in **A** and **B** are the combined signal from endogenous eEF2 + plasmid His-eEF2. Anti-Pgk1was used as a loading control. **A.** Effect of cochaperones previously shown to affect eEF2 folding, as well as a protein (Dph2) required for eEF2 modification. **B.** Effect of additional Hsp90 cochaperones. **C.** Quantification of the changes in His-eEF2 levels in panels **A** and **B**. The level of His-eEF2 bound to resin in each strain was quantified as described in Materials and Methods. The mean values and standard deviations of three biological replicates, along with a representative of each, are shown. Cochaperones previously linked to eEF2 function are shaded dark gray. Statistical significance was evaluated with GraphPad Prism using Mixed-effects analysis (* P ≤ 0.05; ** P ≤ 0.01; *** P ≤ 0.001). Non-significant values not shown.

Hgh1 was identified as a protein that bridges the interaction of eEF2 with either the Hsp90 or TRiC chaperone systems [33,34]. A physical and genetic network consisting of Cpr7 and Cns1 had been recognized for years [46–48], but only recently were those two cochaperones, along with Hgh1, shown to be involved in regulating eEF2 folding and solubility [34]. The prior studies that examined the connection between the Hsp90 machinery and eEF2 used a system in which eEF2 was expressed under the regulatable *GAL1* promoter [34]. We examined the impact of mutations in the Hsp90 machinery on the levels of soluble His-eEF2 in cell lysates (the Eft2 isoform containing a 6x-His tag) expressed under a constitutive promoter [28]. First, we compared isogenic wild-type (WT) cells, or cells lacking *HGH1* or *CPR7*. *CNS1* is essential, so we tested *cns1* cells expressing WT *CNS1* or the temperature-sensitive mutation *cns1-G90D*, which alters a residue in an essential domain [34,49]. As shown in **Fig 1**, isolation of 6xHis-eEF2 using nickel resin results in one predominant band that migrates at the expected size. In agreement with the prior study [34], deletion of *HGH1* or *CPR7*, or mutation of *CNS1*, resulted in significantly lower levels of the introduced His-eEF2 bound to nickel resin and also resulted in lower levels of eEF2 in cell lysates, which is a combination of endogenous and introduced His-eEF2. Reduced levels in our assay may be due either to insolubility of His-eEF2 or misfolding and subsequent degradation of His-eEF2. In contrast, the level of His-eEF2 was not significantly affected by deletion of *DPH2*, one of the genes required for the diphthamide modification [50], indicating that eEF2 folding is not dependent on the modification. A prior study found that deletion of *STI1* did not affect eEF2 levels [20]. We confirmed and extended that study, demonstrating that levels of His-eEF2 were not significantly decreased in cells lacking the cochaperones encoded by *STI1*, *CPR6*, *AHA1*, or *SBA1*. Together, these results support prior studies that suggest that a subset of Hsp90 cochaperones are required for eEF2 stability and/or solubility [20,34].

Overexpression of eEF2 was shown to have toxic effects on growth of cells lacking *CPR7* or with reduced levels of *CNS1* [34]. We similarly found that overexpression of eEF2 significantly reduced the growth of *cpr7 and cns1-G90D* cells at 30°C **(S1 Fig)**. Surprisingly, negative growth defects were not observed in cells lacking *HGH1*. Overexpression of eEF2 did not have significant negative effects on growth of cells lacking *DPH2* or the cochaperones *STI1*, *CPR6*, *AHA1*, or *SBA1*. Thus, except for cells lacking *HGH1*, there was strong correlation between the level of His-eEF2 and negative effects of eEF2 overexpression. The reason for this is unknown. Schopf *et al*. [34] showed that about 20% of eEF2 remained soluble after *CPR7* deletion or *CNS1* knockdown, but about 40% of eEF2 remained soluble after *HGH1* deletion [34], so it is possible that the amount of insoluble eEF2 impacts the level of negative effects on growth.

### Hsp90 mutants have varied effects on His-eEF2 function

**Fig 2:**
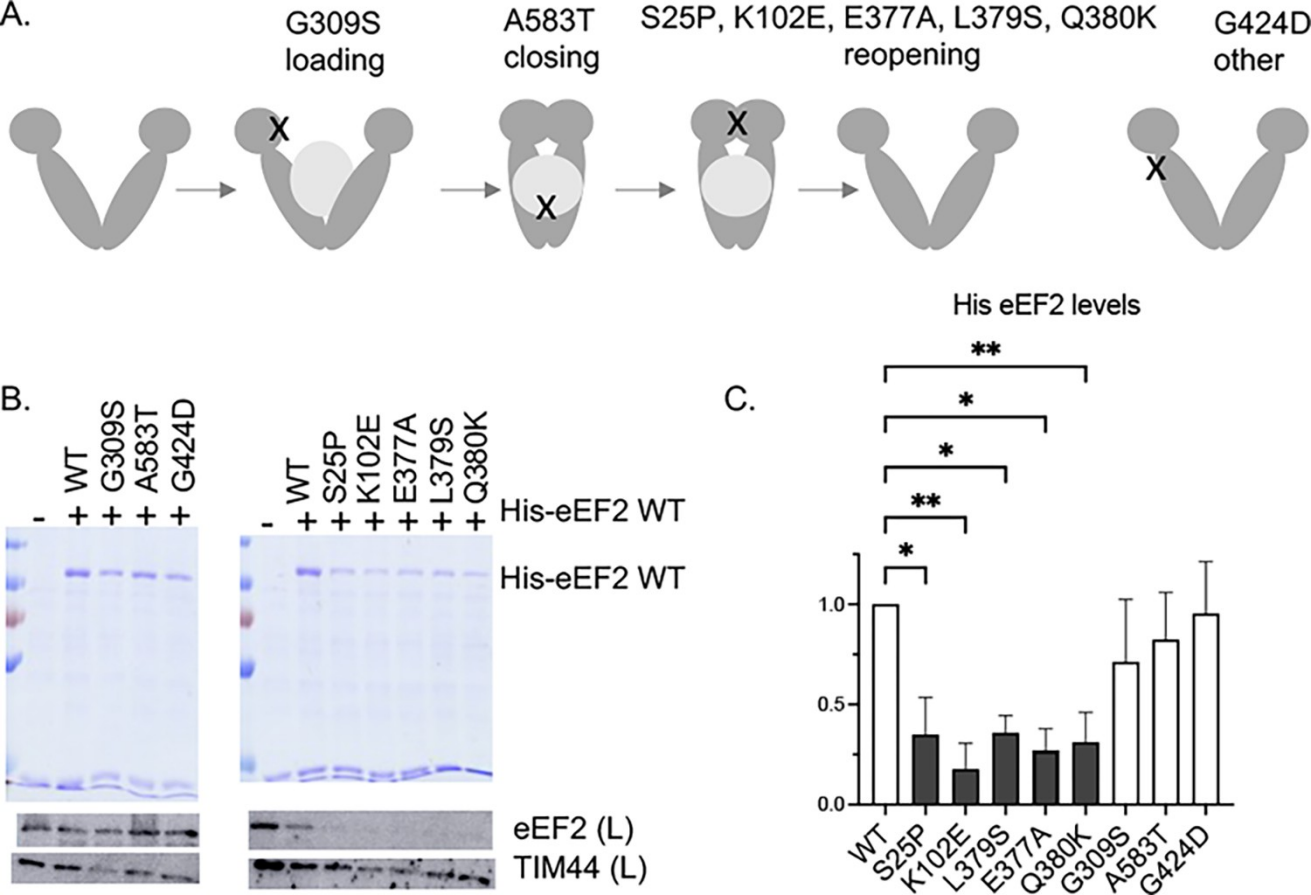
Effect of Hsp90 mutation on 6x-His eEF2 levels. **A.** Schematic of the different Hsc82 mutants and the approximate location of the mutants. The loading mutants disrupt interaction of Hsp70 and Hsp90 during client loading and the closing mutants disrupt formation of the closed state. The reopening mutants cluster in regions required for ATP binding and/or hydrolysis. The G424D mutation is in a distinct region. Additional details about the Hsc82 mutants are listed in **S2 Table**. **B.** Wild-type or mutant Hsp90 (the Hsc82 isoform) was expressed in strain JJ816 (*hsc82hsp82*)**.** His-eEF2 was isolated using nickel resin. Proteins bound to the nickel resin were separated using SDS-PAGE and visualized using stained gels (top). Levels of eEF2 were also detected using immunoblot analysis of whole cell lysates (below). eEF2 levels in the immunoblot are the combined signal from endogenous eEF2 plus plasmid His-eEF2. Anti-TIM44 was used as a loading control. Lane 1: empty vector (YCplacIII). **B.** The level of His-eEF2 bound to resin in each strain was quantified as described in Materials and Methods. The mean values and standard deviations of biological replicates, along with a representative of each, are shown. Hsc82 mutants in the reopening category are shaded in dark gray. Statistical significance was evaluated with GraphPad Prism using Mixed-effects analysis (* P ≤ 0.05; ** P ≤ 0.01). Non-significant values (P ≥ 0.05 not shown).

We next compared levels of His-eEF2 in cells expressing either WT or mutant forms of Hsc82. Each of the previously described mutants displays growth defects at elevated temperature (37°C), but the mutants affect distinct steps in the folding pathway and are differentially affected by overexpresssion of the *HCH1* (not *HGH1*) cochaperone [35,36] **(**summarized in **S2 Table)**. In a simplified model of the folding pathway **(Fig 2A)**, client is loaded onto Hsp90, and then Hsp90 progresses to the closed state. Upon ATP hydrolysis and client release, Hsp90 cycles back to the open complex. One mutant in this study disrupts the loading step (G309S), one disrupts the closing step (A583T), and five mutants disrupt the reopening step (S25P, K102E, E377A, L379S, and Q380K). Another mutant, G424D, causes temperature-sensitive growth but does not appear to affect the steps above. As shown in **Fig 2B and 2C**, His-eEF2 levels were significantly affected only in strains expressing the reopening mutants (S25P, K102E, Q380K, L379S, and E377A). The S25P, K102E, and Q380K mutations also resulted in the lowest relative levels of Eft1/2 in a recent analysis by our laboratory of the effects of Hsp90 mutation on a proteome-wide scale [37]. The amino acids altered in the reopening mutants cluster near regions important for regulation of ATP interaction or hydrolysis [51–53]. Our demonstration that the reopening mutants have the strongest effect on accumulation of His-eEF2 is the first demonstration that mutants in the reopening group have the strongest effect on folding of a specific client.

We next tested whether overexpression of eEF2 in yeast expressing Hsp90 mutants resulted in negative growth defects, similar to those observed in strains lacking *CPR7* or expressing *cns1-G90D*. The effect was readily apparent upon transformation of plasmids into the strains. Wild-type Hsp90, G309S, A583T, or G424D cells expressing either the control plasmid or the His-eEF2 grew similarly after three days. However, cells expressing reopening mutants (S25P, K102E, E377A, L379S or Q380K) exhibited severe growth defects in the presence of His-eEF2 **(S2 Fig)**. Once colonies appeared, we compared growth using the more sensitive growth assay that uses serial dilutions **(S3 Fig)**. Consistent with our results that the reopening mutants cause the strongest eEF2 defects, strong growth defects were observed in cells expressing the reopening mutants**.** However, growth defects were also observed in cells expressing G309S and A583T. The only mutant that did not show a significant growth defect upon eEF2 overexpression was G424D. The G424D mutant had limited effects in our proteomics analysis [37] and G424D did not exhibit defects in activity of other clients that were observed in the loading and closing mutants [35]. At this time, the client defects responsible for the temperature-sensitive growth defect of that mutant remains unknown.

### Loss of *HGH1* causes severe growth defects of *hsc82* mutants with the strongest decrease of His-eEF2 levels

**Fig 3:**
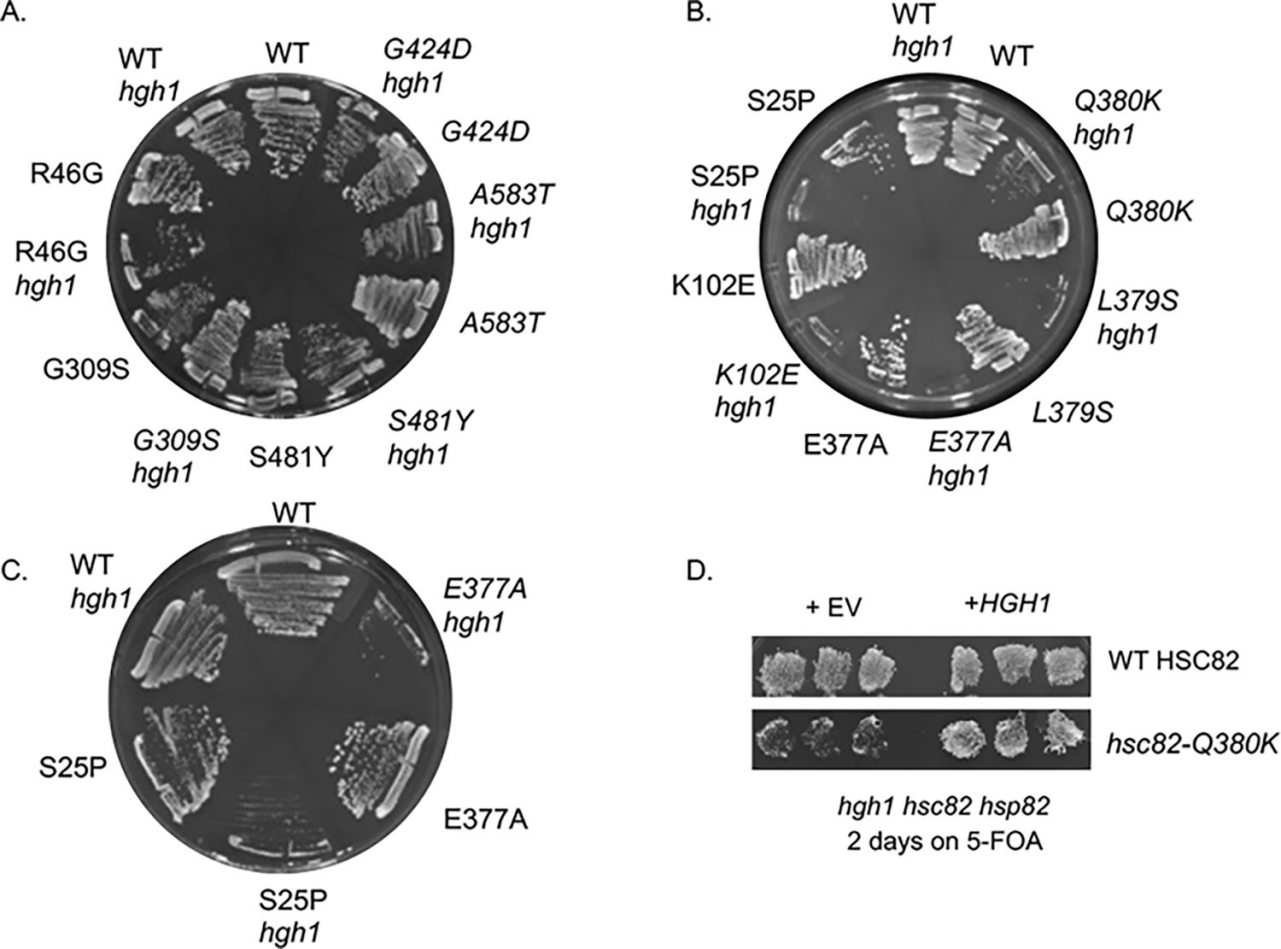
Deletion of *HGH1* enhances the growth defect of some *hsc82* mutant strains. Plasmids expressing either wild-type Hsc82 or the indicated mutant were transformed into isogenic *hsc82hsp82/URA3-HSP82* (JJ816) or *hgh1hsc82hsp82/URA3-HSP82* (JJ1471) strains. **A** and **B**. Strains were streaked onto media containing 5-FOA, which counterselects for the *HSP82* plasmid. Pictures were taken after three days at 30°C. At least three independent assays were conducted, with a representative shown. **C.** Growth of strains in B expressing WT *HSC82*, *hsc82-S25P* or *-E377A* after 5 days on 5-FOA at 30°C. **D**. Strain *hgh1hsc82hsp82/URA3-HSP82* (JJ1471) expressing WT *HSC82* or *hsc82-Q380K* was transformed with a plasmid expressing *HGH1* or empty vector (pRS424). Transformants were patched onto selective media in triplicate, then replicated onto either selective media or plates containing 5-FOA and grown for two days at 30°C.

In previous studies by the Buchner lab [34], loss of *HGH1* did not cause a detectable growth defect, but deletion of *HGH1* enhanced the growth defect of cells lacking *CPR7* [34], reinforcing evidence that the two proteins have similar *in vivo* functions. We next examined the effect of *HGH1* deletion on growth defect of cells expressing *hsc82* mutants **(Fig 3A and 3B)**. We included additional previously described mutants in each group in these assays to broaden our understanding of the phenotypes of the different groups (**S2 Table** [35–37]). R46G and G309S exhibit decreased Hsp70 interaction and are in the ’loading’ group [12,35]. S481Y and A583T exhibit reduced interaction with the Sba1 and Cpr6 cochaperones in the presence of AMP-PNP and are in the ’closing’ group [35,54]. Isogenic *hsc82hsp82* or *hgh1hsc82hsp82* strains that harbor a *URA3*-marked plasmid expressing *HSP82* were transformed with plasmids expressing forms of Hsc82. Transformants were plated onto media containing 5-FOA, which counterselects for the *HSP82* plasmid. As shown in **Fig 3A**, strains expressing WT Hsc82 grow equally well on 5-FOA in the presence or absence of *HGH1*. Similar sized colonies also appeared in cells expressing the mutants in the loading (G309S) and closing group (A583T) in the presence or absence of *HGH1* after three days on 5-FOA **(Fig 3A)**. In contrast, strains expressing the reopening mutants all grew very poorly in the absence of *HGH1* after the same amount of time on 5-FOA **(Fig 3B)**. No colonies appeared in *hgh1* strains expressing S25P or E377A after three days, but small colonies were visible after five days **(Fig 3C)**. To validate the *hgh1hsc82hsp82* strain, empty vector or a plasmid overexpressing *HGH1* was transformed into the strains expressing either WT Hsc82 or Q380K. As shown in **Fig 3D**, the presence of *HGH1* restored growth in the presence of 5-FOA. To quantify the growth defects and minimize effects due to differences in the amount of cells struck out on the plate, colonies were picked from the 5-FOA plates in **Fig 3A and 3B**, grown overnight, normalized, and serially diluted onto rich media. We did not test the reopening mutants S25P and E377A in this assay due to the severe growth defect in the absence of *HGH1***.** As expected, the remaining reopening mutants, K102E, L379S, and Q380K, exhibited strong growth defects in cells lacking *HGH1* **(S4 Fig).** Using this more sensitive assay, some effects negative effects were also observed in cells expressing G309S and A583T. However, the growth of cells expressing G424D was not significantly affected by loss of *HGH1*, consistent with other results showing that this mutant has distinct phenotypes. Thus, Hsc82 mutants differ in their effect on eEF2 functions, as evidenced by reduced accumulation of His-EF2, negative effects of EF2 overexpression, and growth defects in the absence of *HGH1*. In each case, the reopening mutants (S25P, K102E, Q380K, L379S, and E377A) had the strongest effects. The reason that the reopening mutants have the strongest defects related to eEF2 is unknown. The most likely explanation is that the reopening mutants may not be able to form complexes with Hgh1 and/or eEF2. The Buchner lab did not detect a direct interaction between purified Hgh1 and purified Hsp90 or purified eEF2 and purified Hsp90. However, Hsp90 was able to directly bind Cns1 or a complex consisting of Hgh1, Cns1, and eEF2 [34].

### Mutations in eEF2 have distinct effects on eEF2 folding

**Fig 4:**
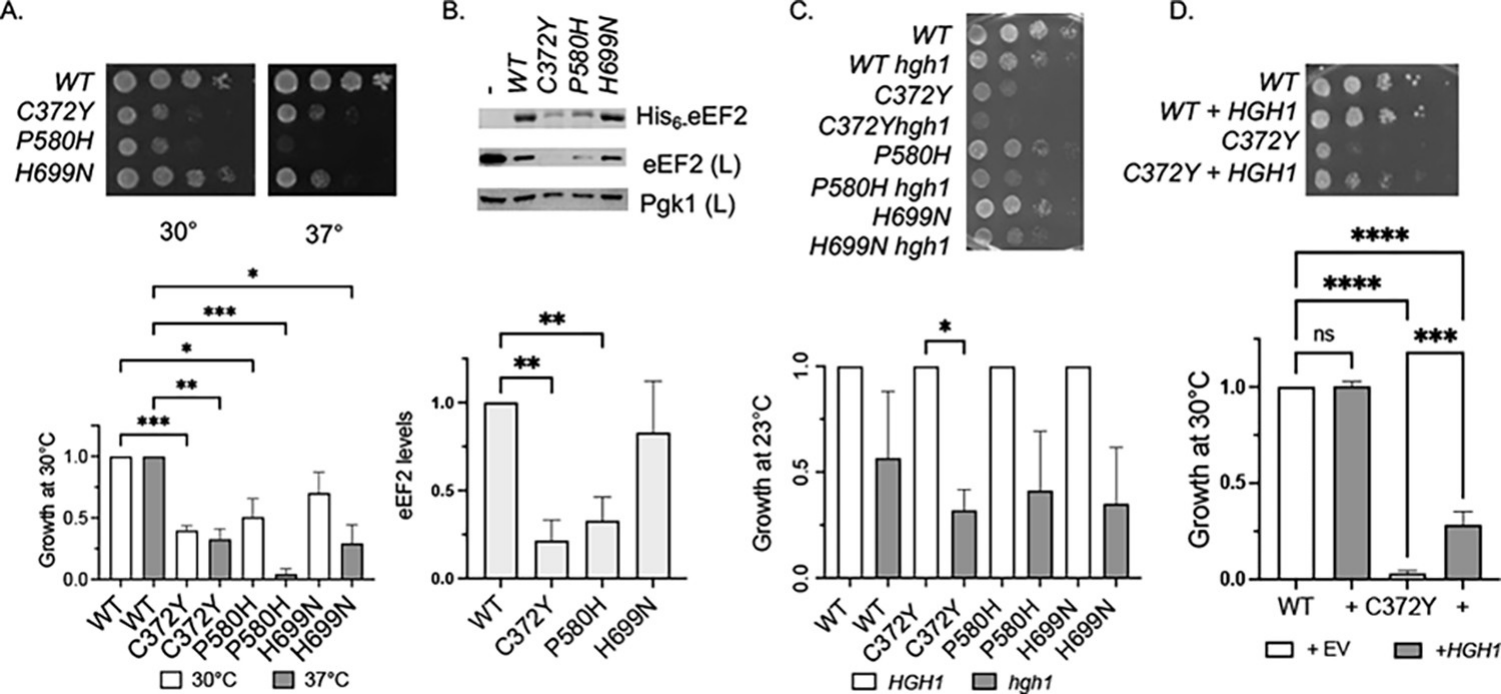
Effect of eEF2 mutation on growth and steady state level. **A**. Growth of wild-type or mutant His-EF2 in *eft1eft2* strain (JJ1472). Strains expressing indicated His-eEF2 plasmid were grown overnight, then serially diluted 10-fold and grown for two days at the indicated temperature. Growth of *eft1eft2* strains expressing each eEF2 mutant was normalized to the growth of the cells expressing WT eEF2 strain. **B.** His-eEF2 was isolated using nickel resin and analyzed by SDS-PAGE followed by staining with Coomassie Blue and immunoblot analysis. Lane 1 is a control that lacks His-tagged protein (JJ1472). The level of His-eEF2 bound to resin in each strain was quantified as described in Materials and Methods. The mean values and standard deviations of three biological replicates, along with a representative of each, are shown. **C**. Growth of WT or mutant eEF2 in *eft1eft2* strain (JJ1472) or *eft1eft2hgh1* strain (JJ1481) after 3 d at 23°C. Growth was normalized to growth of WT or mutant eEF2. **D**. Growth of WT or mutant eEF2 in *eft1eft2* strain in the presence of empty vector (pRS424) or *HGH1* (prs424-*HGH1*) after 2 d on selective media at 30°C. The growth defect on selective media is stronger than when grown on rich media (YPD, in **A**). Growth was normalized to growth of WT eEF2 in the presence of empty vector (EV). Three biological replicates were obtained, with representative pictures of each shown. Statistical significance was evaluated with GraphPad Prism using one way ANOVA. (* P ≤ 0.05; ** P ≤ 0.01; *** P ≤ 0.001; **** P ≤ 0.0001).

eEF2 is a large multidomain protein with five domains. Domain I binds GTP, domain III binds Hgh1 and domain IV contains the diphthamide modification, which occurs on His699 [26,34]. A yeast model system previously examined the effects of two eEF2 mutations that mimic human eEF2 mutations associated with neurodevelopmental disorders, C372Y and P580H. C372Y alters a residue in domain II and P580H is in domain IV, close to H699 [28,29]. We expressed His-tagged versions of those mutants, as well as H699N, which blocks the modification, in an *eft1eft*2 strain isogenic to our other strains (**Fig 4A**). All three of these mutations resulted in growth defects at elevated temperature, with C372Y and P580H also causing reduced growth at the optimal growth temperature of 30°C. To determine the effect of mutation on folding, WT and mutant His-eEF2 were isolated out of yeast as described above. As shown in **Fig 4B**, His-eEF2 WT and H699N accumulated to similar levels, consistent with a prior analysis [55], but the C372Y and P580H alterations resulted in significantly reduced levels of His-eEF2, suggesting that misfolding of eEF2 contributes to the human disorders associated with these variants.

Since Hgh1 directly interacts with eEF2 and enhanced growth defects were observed upon combination of *hsc82* mutants and *HGH1* deletion, we constructed isogenic *eft1eft2* and *eft1eft2hgh1* strains. The loss of *HGH1* reduced the growth of cells expressing all mutants, with a significant reduction in cells expressing C372Y **(Fig 4C**). Further, overexpression of *HGH1* partially rescued the growth defect of cells expressing C372Y **(Fig 4D)**. In addition to providing new evidence that Hgh1 has a role in folding of eEF2, this suggests that overexpression of Hgh1 may help rescue defects of human eEF2 caused by the homologous mutation. Results from the Buchner lab indicate that domain III of eEF2 binds Hgh1 [34], and it is possible that stabilization of domain III may help overcome folding defects in the nearby domain that arise due to the C372Y mutation.

### Effects on eEF2, Hsp90, or cochaperone mutation on sensitivity to diphtheria toxin

**Fig 5:**
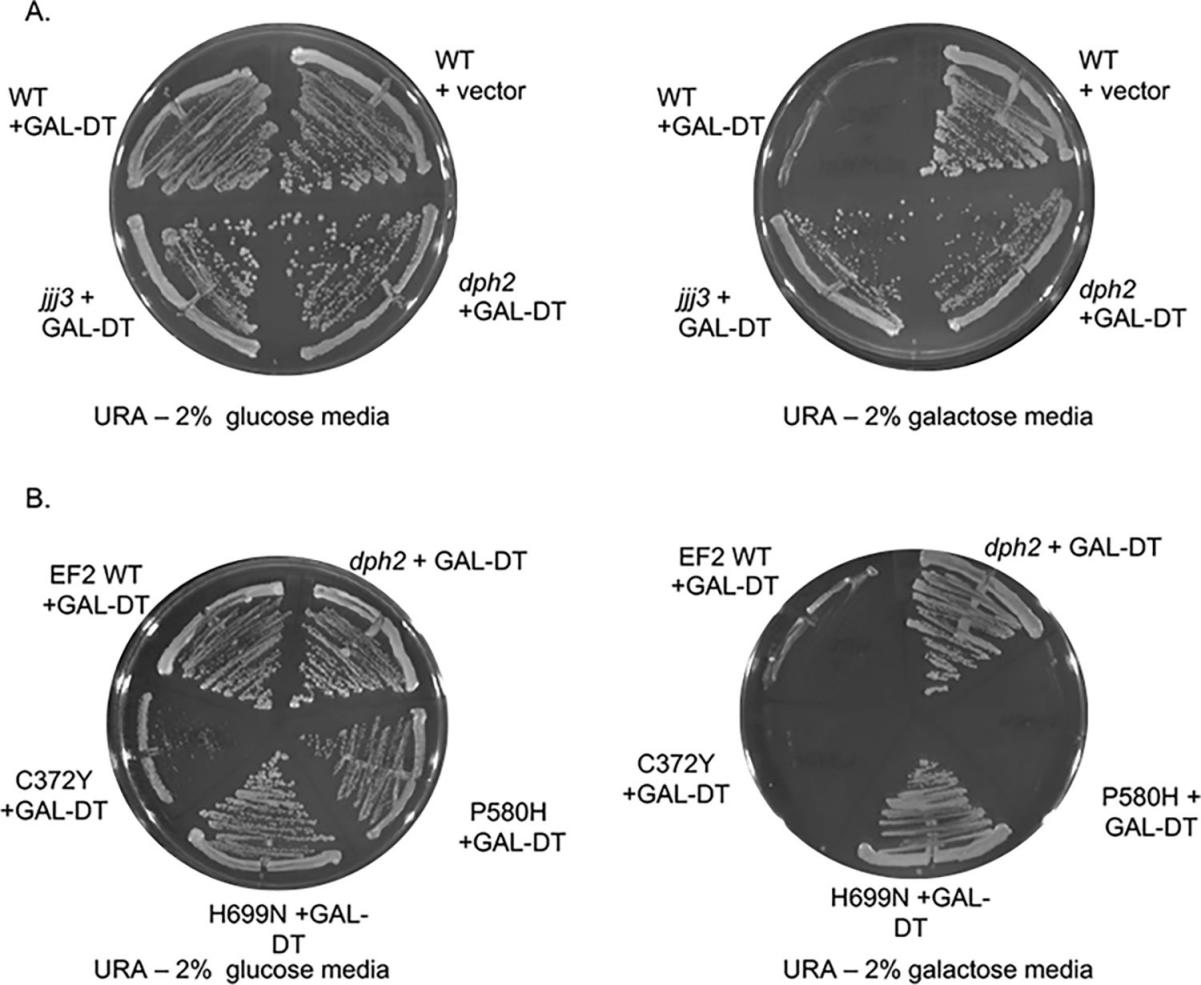
Effect of eEF2 mutation sensitivity to diphtheria toxin. **A**. WT cells (762) or *dph2*, or *jjj3* strains were transformed with empty vector (-, pB656) or pLMY101, which expresses the catalytic domain of DT under the *GAL1* promoter [41]. Cells were struck out onto selective (- uracil) plates containing 2% glucose (as a control) or 2% galactose and grown for four days at 30°C. **B.** Strain 1472 (*eft1eft2*) expressing wild-type or mutant His-EF2 was transformed with plasmid pLMY101. A *dph2* strain harboring pLMY101 was used as a control. Cells were struck out onto selective (- uracil) plates containing 2% glucose (as a control) plates containing 2% galactose and grown for four days at 30°C. Three biological replicates were obtained, with representative pictures of each shown.

In wild-type yeast, the presence of diphtheria toxin (DT) results in irreversible ADP ribosylation of diphthamide, inactivation of EF2, and cell death [41]. To test the effect of mutations on the diphthamide modification, cells were transformed with a plasmid that expresses the catalytic domain of DT in the presence of galactose (GAL-DT). Wild-type (WT) cells expressing empty vector grow similarly on selective media containing glucose (Ura- glucose) or galactose media (Ura- galactose)**,** but WT cells expressing GAL-DT die on galactose **(Fig 5A**). As controls, we tested an isogenic strain lacking *DPH2*, which encodes an enzyme required for the first step of diphthamide biosynthesis [50,56], as well as a non-isogenic strain lacking *JJJ3/DPH4*, which encodes another protein required for the modification [57]. As expected, both strains grew in the presence of galactose despite the presence of DT.

We then tested the effect of DT expression on cells expressing WT or mutant eEF2. As expected, the H699N alteration, which prevents the modification, enabled cells to grow in the presence of DT **(Fig 5B)**. The other mutants, C372Y and P580H, both died in the presence of DT, suggesting they do not impact the modification. Of note, cells expressing C372Y consistently grew poorly on media lacking uracil, for unknown reasons. As an additional test, we reduced the amount of galactose in the media from the standard 2% to 1%, since one study reported that cells lacking *DPH7*, which encodes one of the genes required for the modification, were unable to grow in the presence of 2% galactose but were able to grow when galactose levels were reduced [58]. However, that did not affect growth of cells expressing P580H and C372Y **(S5 Fig).**

**Fig 6:**
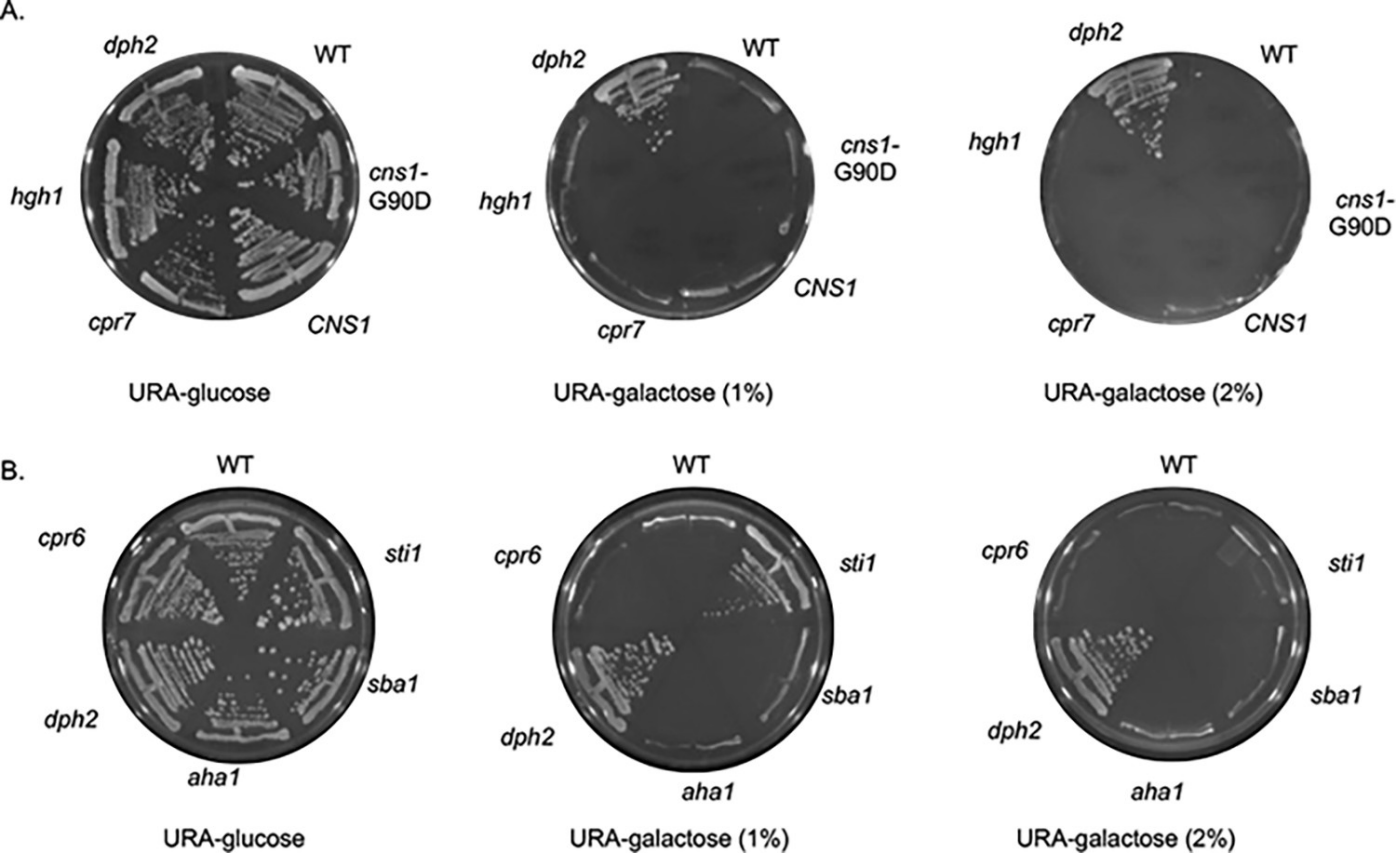
Effect of cochaperone deletion or alteration on sensitivity to diphtheria toxin. **A.** WT cells (JJ762), cells lacking *HGH1* (JJ1465), *CPR7* (JJ1115) or *DPH2* (JJ1449) or a *cns1* disruption strain (JJ21) expressing WT *CNS1* or *cns1-G90D* were transformed with plasmid pLMY101 and struck out onto selective (- uracil) plates containing 2% glucose (as a control), 1% raffinose and 1% galactose, or 2% galactose and grown for four days at 30°C. **B.** WT cells (JJ762), cells lacking *CPR6* (JJ1138), *STI1* (JJ623), *AHA1* (JJ73), *SBA1* (JJ543), or *DPH2* (JJ1449). Three biological replicates were obtained, with representative pictures of each shown.

**Fig 7:**
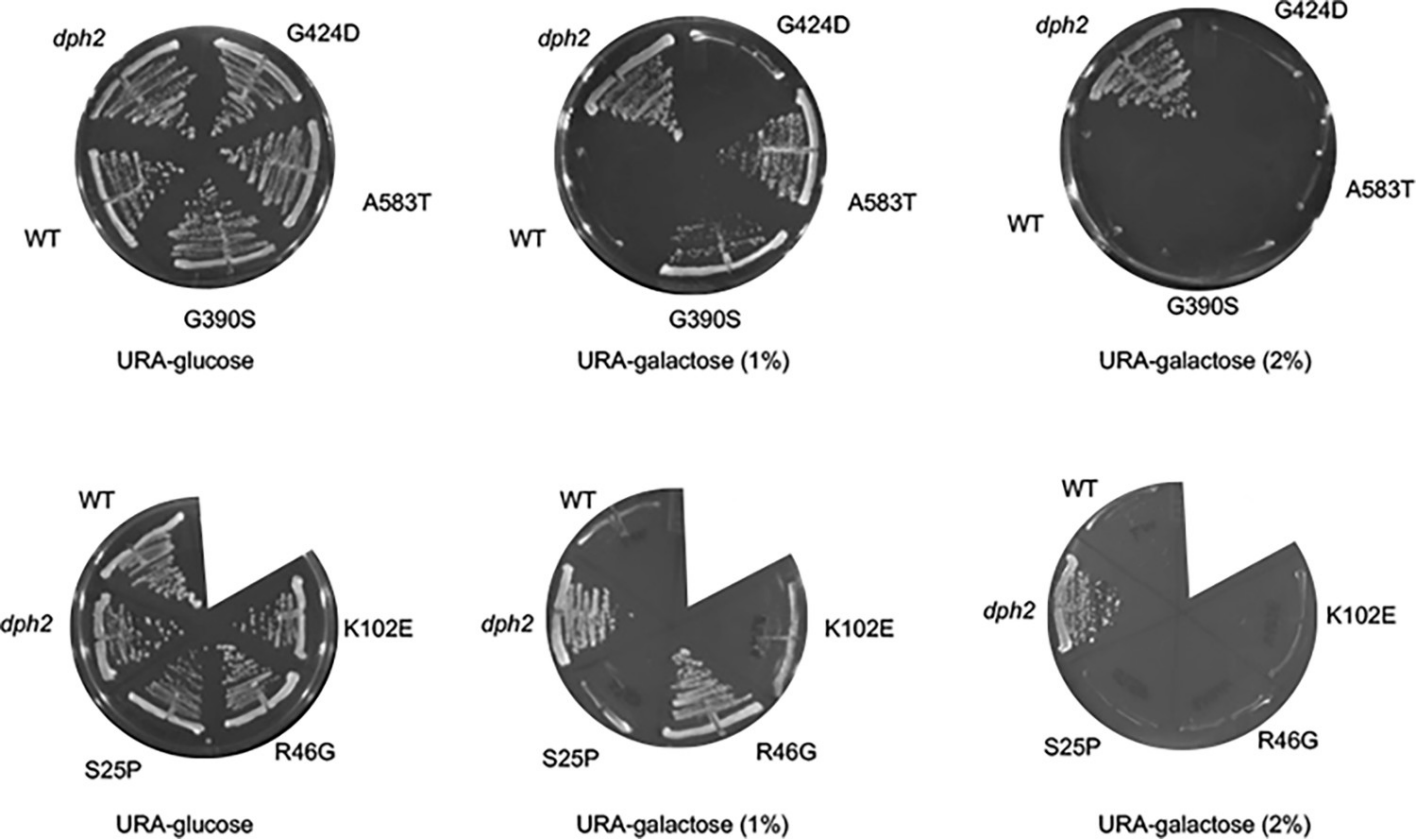
Effect of Hsc82 cochaperone deletion on sensitivity to diphtheria toxin. Strain JJ117 expressing WT or mutant Hsc82 was transformed with plasmid pLMY101 and struck out onto selective (- uracil) plates containing 2% glucose (as a control), 1% raffinose and 1% galactose, or 2% galactose and grown for four days at 30°C. A *dph2* strain harboring pLMY101 was used as a control. Three biological replicates were obtained, with representative pictures of each shown.

We then tested cochaperone mutations. As shown in **Fig 6A**, strains lacking *CPR7* or *HGH1* or expressing *cns1-G90D* exhibited wild-type sensitivity to DT in the presence of 1 or 2% galactose. Surprisingly, deletion of *STI1*, but not deletion of *SBA1*, *CPR6*, or *AHA1* provided resistance to DT under conditions of reduced galactose (1%) **(Fig 6B)**. In addition, cells expressing Hsc82-R46G, G309S, or A583T were also able to grow in the presence of 1% galactose, while cells expressing other Hsc82 mutants, including S25P, K102E, and G424D, were unable to grow **(Fig 7)**. This suggests that some Hsp90 or cochaperone mutations affect the process or efficiency of the diphthamide modification.

## Discussion

The molecular chaperone Hsp90 is an important drug target due to its role in chaperoning proteins required at multiple stages of cancer progression and proteins that promote fungal infections [59–61]. However, inhibitors that target the ATP-binding site have negative effects that limit clinical use [62,63]. A major question in the field is how to achieve selective inhibition of Hsp90, which impacts the function of 15–20% of the proteome [1–4]. We developed a robust system to study the impact of mutations that target distinct steps in the Hsp90 folding pathway in the genetically tractable *S*. *cerevisiae* [35–37]. Hsp90 mutants that disrupt early steps in the folding cycle had the largest effect on ’classic’ clients such as protein kinases. Here we identify the first client defect that is strongest in the ’refolding’ mutants. Moreover, we provide evidence that defects in classes of Hsp90 mutants are linked to subsets of cochaperones. This suggests that there may be distinct versions of the Hsp90 folding pathway that chaperone different types of clients. A better understanding of the basis of these differences may lead to selective inhibitors with fewer negative effects.

**Fig 8:**
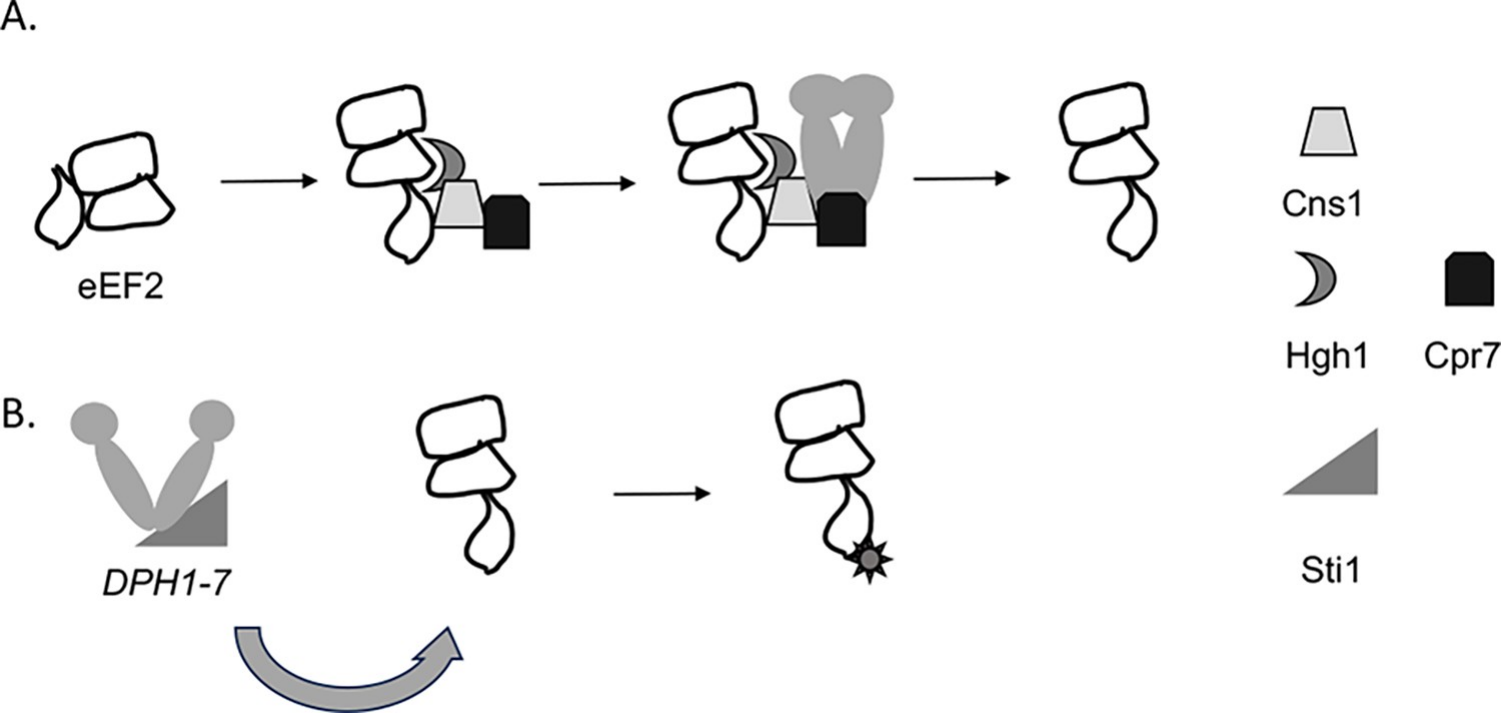
Model of Hsp90 and cochaperone interaction with eEF2. A. Folding of eEF2 is assisted by Hgh1, Cns1 and Cpr7. Hsp90 binds the eEF2 cochaperone complex, further assisting folding. Reduced function of Hsp90 or these cochaperones results in inefficient folding and/or aggregation of eEF2. B. Hsp90 and Sti1 promote maturation of proteins required for efficient processing of the diphthamide modification, such as Dph6 and Dph7. Reduced function of Hsp90 and/or Sti1 may result in inefficient modification of eEF2, resulting in resistance to DT.

eEF2 is an essential protein with critical roles in ribosome translocation. As shown in the model in **Fig 8**, our results suggest two separate roles for Hsp90 and cochaperones. Along with Hgh1, Cns1, and Cpr7, Hsp90 promotes proper folding of eEF2. The absence of this function causes eEF2 to misfold and, in some cases, aggregate, resulting in growth defects due to loss of essential functions. In addition, Hsp90 and Sti1 appear to have a role in promoting the diphthamide modification. At least seven genes (*DPH1-DPH7* in yeast) cooperate in a multistep pathway to generate the fully modified form of eEF2 [27,64]. A prior study identified a link between yeast Hsp90 and *DPH6* or *DPH7*, suggesting that Hsp90 may be required for those proteins to work properly [4]. Interestingly, one of the proteins required for the diphthamide modification, Dph4/Jjj3 contains a J domain, which mediates interaction with Hsp70 molecular chaperones [57,65]. Other J proteins, such as Ydj1 are known to be required for Hsp90 clients [66–68]. Further studies are required to determine if eEF2 isolated from Hsp90 mutant strains have reduced ability to fully modify the H699 residue. **S2 Table** summarizes our results, which suggest at least three possible fates of eEF2 upon alteration of Hsc82: reduced accumulation with little or no impact on sensitivity to DT (the reopening mutants); reduced sensitivity to DT with some impact on steady-state levels (loading and closing mutants); or little or no effect on either accumulation or sensitivity to DT (G424D, ’other’). This is the first demonstration that a single Hsp90 client requires two genetically distinct functions of the Hsp90 molecular chaperone machine that differ in cochaperone requirement and the type of Hsp90 mutation that affects function.

Hsc82 mutants that most strongly affect eEF2 steady-state levels (the reopening mutants) show strong synthetic growth defects when combined with deletion of *HGH1*, which also results in decreased steady-state levels of eEF2. The reopening mutants are predicted to affect ATP hydrolysis, client release and return to the open conformation steps involved in ATP hydrolysis and/or nucleotide exchange **(S2 Table)** [35,36,51,53]. One possibility for the specific effect of the reopening mutants is that efficient folding of eEF2 requires a specific rate of ATP hydrolysis and/or nucleotide exchange, and that any mutant that delays ATP hydrolysis, ADP release, or alters the timing of the conformational cycle disrupts function [69]. Although a previous study in the Bolon lab showed that the rate of ATP hydrolysis did not appear to be a factor in affecting specificity for GR or v-src [70], this has not been tested for eEF2. Another option is that the reopening mutants disrupt a specific conformation of Hsp90 that is required for eEF2 or Hgh1 interaction. *CNS1* was isolated as a multicopy suppressor of the *hsp82-E381K* allele, which contains an alteration in the same flexible loop as many of the reopening mutants [36,71]. Purified Hsp90 bound Cns1 directly, as well as a complex consisting of Hgh1, Cns1, and eEF2 [34]. It is possible that Cns1 interaction stabilizes the conformation of Hsp90 required for formation of the Hgh1-Cns1-eEF2-Hsp90-complex. A direct interaction between Hgh1 and purified Hsp90 was not detected in studies by the Buchner lab. However, eEF2 was found to cross-link a site in the middle domain of Hsp90 using the artificial amino acid Bpa [1]. Thus, the Hsp90-eEF2 interaction may be transient and not detectable without stabilization through crosslinkers. Our efforts to reproducibly detect and quantify differences in how Hsp90 mutation affects interaction with eEF2 or Hgh1 were unsuccessful, likely due to differences in the abundance of eEF2, the transient nature of the of the interaction and/or background binding of Hsp90 to resin used during pulldown assays.

The Hsp90 folding cycle is complex and may vary from client to client [14–18], but one consistent feature is the presence of Hsp90-client-cochaperone complexes. During Hsp90-assisted folding of the GR, GR bound to Hsp70 is targeted to Hsp90 with assistance from Hop/Sti1. Sti1 directly participates in client loading, mutation of *STI1* results in defects in GR and v-src activity, and Hsc82 mutations in the loading and closing group are inviable in the absence of *STI1* [12,35,72,73]. We previously identified similar connections between Hsp90, Sti1, and the essential client Utp21, which functions in ribosome biogenesis. A mutation in *UTP21* that results in mild growth defects on its own results in lethal or severe growth defects when combined with deletion of *STI1 or* when combined with Hsc82 mutants in the loading and closing group [35,74]. Our hypothesis is that the mutated form of Utp21 is particularly reliant on the ability of Sti1 to load clients onto Hsp90, and that the combination of two separate mutations that affect this process results in loss of the essential function of Utp21 in ribosome biogenesis. Similarly, the cochaperone Cdc37 directly binds protein kinases and targets them to Hsp90 [14]. Activity of v-src kinase was reduced in cells expressing either *cdc37* or Hsc82 mutants in the loading and closing group, and the combination of the *cdc37* mutant and Hsc82 mutants in the loading and closing groups results in inviability [35]. Finally, we previously showed that an alteration of the Sgt1 cochaperone mimics the *in vivo* effects of the Hsc82-W296A, another mutant in our ’other’ category [37,39]. As summarized in **Table 1**, our prior results that indicate a strong correlation between selective effects of Hsp90 mutations and subsets of cochaperones extends to eEF2, the reopening mutants, and Hgh1.

**Table 1:** Functional connections between Hsc82 mutants, cochaperones and clients. A comparison between data in this paper regarding the client eEF2 with prior data. Results from the current study are shaded in gray. (*nd = not determined).

Client function analyzed	eEF2 folding	eEF2 sensitivity to DT	Utp21	Cyr1	v-src kinase
Cochaperone requirement	*HGH1* required to maintain steady state levels of eEF2	Deletion of *STI1* results in enhanced resistance to DT	Deletion of *STI1* results in severe growth defect of cells expressing *utp21* mutation	Mutation in *SGT1* affects cAMP pathway	*CDC37* required for v-src activity
Hsp90 mutant effect on client	Reopening mutants have biggest effect on steady state levels of eEF2	Loading and closing mutants have enhanced resistance to DT	Loading and closing mutants exhibit severe growth defect when combined with *utp21* mutation.	W296A mutation affects cAMP pathway	Loading and closing mutants have biggest effect on v-src activity
Cochaperone-Hsp90 connection	Reopening mutants have severe growth defect in absence of *HGH1*	Loading and closing mutants have severe growth defect in absence of *STI1*	Loading and closing mutants have severe growth defect in absence of *STI1*	n.d.	Loading and closing mutants have severe growth defect when combined with *cdc37* mutation
References	[34], This study	This study, [35]	[35,74,79]	[39]	[35]

Our results, along with the prior analysis of the Hsp90-eEF2 interaction, suggest a role for Hsp90 in translation elongation by modulating the folding of eEF2. Deletion of *CPR7* or alteration of *CNS1* caused increased sensitivity to translational inhibitors, and some *hsc82* mutant strains also have varied sensitivity to hygromycin, supporting a role in translation [34,46]. The eEF2-C372Y mutation also caused sensitivity to translation inhibitors [28]. Studies in the Buchner lab showed that loss of *CPR7* or knockdown of *CNS1* resulted in reduced incorporation of ^35^S-methionine in proteins. They also used polysome run-off experiments to demonstrate that strains with reduced levels of Hgh1, Cpr7 or Cns1 likely have defects in translation elongation or termination [34]. Many of the genes linked to the translational apparatus share a pattern of transcriptional coregulation. Prior studies suggested that Cpr7 and Hgh1 have functions related to translation due to their similar pattern of regulation [75,76]. A recent study demonstrated that a separate Hsp90 client is involved in translation initiation. Hsp90 inhibition reduced the fidelity of start site selection, and evidence suggests that the accumulation of misfolded polypeptides due to translational infidelity triggers the heat shock response mediated by the transcription factor Hsf1 [1]. Loss of *CPR7* or deletion of *HSC82* is known to result in increased basal Hsf1 activity [77]. In yeast, the eEF2 mutations P580H and H699N result in increased -1 frameshifting, and cells expressing P580H exhibited an enhanced stress response [28,29], presumably due to the buildup of mistranslated proteins. Additional studies are required to determine whether cells lacking *CPR7* or expressing the reopening mutants display errors in translational fidelity. However, our studies provide evidence that Hsp90 has critical roles in translation elongation in addition to translation initiation.

Hgh1 was identified as a protein that bridges the interaction of eEF2 with either Cns1 and Hsp90 or with the TRiC chaperone [33,34]. Further studies are required to better understand whether the interaction of Hgh1 and eEF2 with TRiC and Hsp90 represent independent folding pathways or whether TRiC and Hsp90 interact with eEF2 in a sequential manner. We showed that the yeast C372Y mutant, which corresponds to a *de novo* mutation in an individual with neurodevelopmental disorders [28], results in growth defects that were partially rescued by overexpression of *HGH1*, suggesting that it may be possible to alleviate folding defects of some forms of mutated eEF2. Further studies are needed to determine the scope of Hgh1 functions. In yeast, deletion of the genes in the diphthamide synthesis pathway has mild effects on growth, but in humans, alteration of the pathway results in developmental or neurological disorders [25,65]. Further studies are also required to understand the connection between Hsp90 and susceptibility to DT. Interestingly, this is not the first connection between Hsp90 cochaperones and DT. A prior study found that Hsp90, Hsp70, and cochaperones such as Cyp40, FKBP51, and FKBP52, facilitate the transport of diphtheria toxin into human cells [78]. This highlights the vast cellular roles of Hsp90 in mediating cellular functions and the potential impact of modulating Hsp90 and/or cochaperone function in order to treat a variety of human diseases.

## Supporting information

S1 Table Strains used in this study. (DOCX)

S2 Table Information about Hsc82 mutants used in this study. General classification of groups with specific defects listed. Shaded columns represent new data from this study.
(DOCX)

S1 Fig Effect of overexpression of eEF2 in yeast with cochaperone deletion or mutation. **A.** A plasmid expressing wild-type His-eEF2 or empty vector was transformed into the strains shown as in Fig 1. Transformants were grown overnight in selective media, serially diluted 10-fold and grown on selective media for 2 days at 30°C. **B.** Growth of biological replicates expressing eEF2 normalized to cells expressing empty vector, with representative pictures of each shown. Cochaperones previously shown to be required for eEF2 folding, are shaded in dark gray. Statistical significance was evaluated with GraphPad Prism using Mixed-effects analysis (* P ≤ 0.05). Non-significant values (P ≥ 0.05 not shown).
(TIF)

S2 Fig Overexpression of eEF2 causes negative effect in cells expressing reopening mutants. A plasmid expressing wild-type His-eEF2 (bottom) or empty vector (top) was transformed into *hsc82hsp82* strains expressing either wild-type Hsc82 or the indicated mutant. Pictures were taken after growth on selective media for 2 or 3 days at 30°C.
(TIF)

S3 Fig Effect of overexpression of eEF2 on growth of cells containing mutations in Hsp90. A plasmid expressing wild-type His-eEF2 or empty vector was transformed into the strains as in S2 Fig. Transformants were grown overnight in selective media, serially diluted 10-fold and grown on selective media for 2 days at 30°C. Growth of biological replicates expressing EF2 was normalized to cells expressing empty vector, with representative pictures of each shown. Hsc82 mutants in the reopening category are shaded in dark gray. Statistical significance was evaluated with GraphPad Prism using Mixed-effects analysis (* P ≤ 0.05; ** P ≤ 0.01; *** P ≤ 0.001). Non-significant values (P ≥ 0.05 not shown).
(TIF)

S4 Fig Quantification of growth of *hsc82* mutants in the presence or absence of *HGH1*. Colonies were picked from the 5-FOA plates in Fig 2, grown overnight in rich media, serially diluted 10-fold and grown on rich media (YPD) for 2 days at 30°C. Growth of *hgh1hsc82 hsp82* strains expressing each *hsc82* mutant was normalized to the growth of the same mutant in an *hsc82hsp82* strain. Three biological replicates were obtained, with representative pictures of each shown. Hsc82 mutants in the reopening category are shaded in dark gray. Statistical significance was evaluated with GraphPad Prism using Mixed-effects analysis (* P ≤ 0.05). Non-significant values (P ≥ 0.05 not shown).
(TIF)

S5 Fig Effect of altered galactose concentration. Strain 1472 (*eft1eft2*) expressing wild-type or mutant His-EF2 was transformed with plasmid pLMY101. Strains were grown on selective (- uracil) plates containing 0.5%, 1%, or 2% galactose or 2% glucose (as a control) and grown for four days at 30°C. As needed, raffinose was added so that the total of raffinose plus galactose was 2%. Three biological replicates were obtained, with representative pictures of each shown.
(TIF)

S1 Source Data The file contains the quantification data needed for the graphs. (XLSX)

## References

[1] Kolhe JA, Babu NL, Freeman BC. The Hsp90 molecular chaperone governs client proteins by targeting intrinsically disordered regions. Mol Cell. 2023. Epub 20230601. doi: 10.1016/j.molcel.2023.05.021 . 37295430 PMC10297700

[2] Girstmair H, Tippel F, Lopez A, Tych K, Stein F, Haberkant P, et al. The Hsp90 isoforms from S. cerevisiae differ in structure, function and client range. Nat Commun. 2019;10(1):3626. Epub 2019/08/11. doi: 10.1038/s41467-019-11518-w ; PubMed Central PMCID: PMC6689086. 31399574 PMC6689086

[3] Zhao R, Davey M, Hsu YC, Kaplanek P, Tong A, Parsons AB, et al. Navigating the chaperone network: an integrative map of physical and genetic interactions mediated by the hsp90 chaperone. Cell. 2005;120(5):715–27. PubMed doi: 10.1016/j.cell.2004.12.024 . 15766533

[4] McClellan AJ, Xia Y, Deutschbauer AM, Davis RW, Gerstein M, Frydman J. Diverse cellular functions of the hsp90 molecular chaperone uncovered using systems approaches. Cell. 2007;131(1):121–35. PubMed doi: 10.1016/j.cell.2007.07.036 . 17923092

[5] Franzosa EA, Albanese V, Frydman J, Xia Y, McClellan AJ. Heterozygous yeast deletion collection screens reveal essential targets of Hsp90. PLoS One. 2011;6(11):e28211. Epub 2011/12/06. doi: 10.1371/journal.pone.0028211 [pii]. ; PubMed Central PMCID: PMC3227642. 22140548 PMC3227642

[6] Schopf FH, Biebl MM, Buchner J. The HSP90 chaperone machinery. Nat Rev Mol Cell Biol. 2017;18(6):345–60. doi: 10.1038/nrm.2017.20 . 28429788

[7] Alvira S, Cuellar J, Rohl A, Yamamoto S, Itoh H, Alfonso C, et al. Structural characterization of the substrate transfer mechanism in Hsp70/Hsp90 folding machinery mediated by Hop. Nat Commun. 2014;5:5484. doi: 10.1038/ncomms6484 . 25407331

[8] Lopez A, Dahiya V, Delhommel F, Freiburger L, Stehle R, Asami S, et al. Client binding shifts the populations of dynamic Hsp90 conformations through an allosteric network. Sci Adv. 2021;7(51):eabl7295. Epub 2021/12/18. doi: 10.1126/sciadv.abl7295 ; PubMed Central PMCID: PMC8682993. 34919431 PMC8682993

[9] Lorenz OR, Freiburger L, Rutz DA, Krause M, Zierer BK, Alvira S, et al. Modulation of the hsp90 chaperone cycle by a stringent client protein. Mol Cell. 2014;53(6):941–53. doi: 10.1016/j.molcel.2014.02.003 . 24613341

[10] Pratt WB, Toft DO. Steroid receptor interactions with heat shock protein and immunophilin chaperones. Endocr Rev. 1997;18(3):306–60. PubMed doi: 10.1210/edrv.18.3.0303 . 9183567

[11] Noddings CM, Wang RY, Johnson JL, Agard DA. Structure of Hsp90-p23-GR reveals the Hsp90 client-remodelling mechanism. Nature. 2022;601(7893):465–9. Epub 2021/12/24. doi: 10.1038/s41586-021-04236-1 ; PubMed Central PMCID: PMC8994517. 34937936 PMC8994517

[12] Wang RY, Noddings CM, Kirschke E, Myasnikov AG, Johnson JL, Agard DA. Structure of Hsp90-Hsp70-Hop-GR reveals the Hsp90 client-loading mechanism. Nature. 2022;601(7893):460–4. Epub 2021/12/24. doi: 10.1038/s41586-021-04252-1 . 34937942 PMC9179170

[13] Noddings CM, Johnson JL, Agard DA. Cryo-EM reveals how Hsp90 and FKBP immunophilins co-regulate the glucocorticoid receptor. Nat Struct Mol Biol. 2023;30(12):1867–77. Epub 20231109. doi: 10.1038/s41594-023-01128-y ; PubMed Central PMCID: PMC10716051. 37945740 PMC10716051

[14] Verba KA, Wang RY, Arakawa A, Liu Y, Shirouzu M, Yokoyama S, et al. Atomic structure of Hsp90-Cdc37-Cdk4 reveals that Hsp90 traps and stabilizes an unfolded kinase. Science. 2016;352(6293):1542–7. doi: 10.1126/science.aaf5023 . 27339980 PMC5373496

[15] Willhoft O, Kerr R, Patel D, Zhang W, Al-Jassar C, Daviter T, et al. The crystal structure of the Sgt1-Skp1 complex: the link between Hsp90 and both SCF E3 ubiquitin ligases and kinetochores. Scientific reports. 2017;7:41626. doi: 10.1038/srep41626 ; PubMed Central PMCID: PMC5282575. 28139700 PMC5282575

[16] Taipale M, Tucker G, Peng J, Krykbaeva I, Lin ZY, Larsen B, et al. A quantitative chaperone interaction network reveals the architecture of cellular protein homeostasis pathways. Cell. 2014;158(2):434–48. doi: 10.1016/j.cell.2014.05.039 ; PubMed Central PMCID: PMC4104544. 25036637 PMC4104544

[17] Biebl MM, Delhommel F, Faust O, Zak KM, Agam G, Guo X, et al. NudC guides client transfer between the Hsp40/70 and Hsp90 chaperone systems. Mol Cell. 2022;82(3):555–69 e7. Epub 2022/01/23. doi: 10.1016/j.molcel.2021.12.031 . 35063133

[18] Clerico EM, Gierasch LM. There are more Hsp90 chaperone mechanisms in heaven and earth, dear reader, than are dreamt of in your philosophy. Mol Cell. 2022;82(8):1403–4. doi: 10.1016/j.molcel.2022.03.040 ; PubMed Central PMCID: PMC9074108. 35452610 PMC9074108

[19] Sahasrabudhe P, Rohrberg J, Biebl MM, Rutz DA, Buchner J. The Plasticity of the Hsp90 Co-chaperone System. Mol Cell. 2017;67(6):947–61 e5. Epub 2017/09/12. doi: 10.1016/j.molcel.2017.08.004 . 28890336

[20] Biebl MM, Riedl M, Buchner J. Hsp90 Co-chaperones Form Plastic Genetic Networks Adapted to Client Maturation. Cell reports. 2020;32(8):108063. Epub 2020/08/28. doi: 10.1016/j.celrep.2020.108063 . 32846121

[21] Dean ME, Johnson JL. Human Hsp90 cochaperones: perspectives on tissue-specific expression and identification of cochaperones with similar in vivo functions. Cell Stress Chaperones. 2021;26(1):3–13. Epub 2020/10/11. doi: 10.1007/s12192-020-01167-0 ; PubMed Central PMCID: PMC7736379. 33037995 PMC7736379

[22] Borkovich KA, Farrelly FW, Finkelstein DB, Taulien J, Lindquist S. hsp82 is an essential protein that is required in higher concentrations for growth of cells at higher temperatures. Mol Cell Biol. 1989;9(9):3919–30. PubMed doi: 10.1128/mcb.9.9.3919-3930.1989 . 2674684 PMC362454

[23] Perentesis JP, Phan LD, Gleason WB, LaPorte DC, Livingston DM, Bodley JW. Saccharomyces cerevisiae elongation factor 2. Genetic cloning, characterization of expression, and G-domain modeling. J Biol Chem. 1992;267(2):1190–7. Epub 1992/01/15. . 1730643

[24] Ghaemmaghami S, Huh WK, Bower K, Howson RW, Belle A, Dephoure N, et al. Global analysis of protein expression in yeast. Nature. 2003;425(6959):737–41. PubMed doi: 10.1038/nature02046 . 14562106

[25] Hawer H, Mendelsohn BA, Mayer K, Kung A, Malhotra A, Tuupanen S, et al. Diphthamide-deficiency syndrome: a novel human developmental disorder and ribosomopathy. Eur J Hum Genet. 2020;28(11):1497–508. Epub 2020/06/25. doi: 10.1038/s41431-020-0668-y ; PubMed Central PMCID: PMC7575589. 32576952 PMC7575589

[26] Pellegrino S, Demeshkina N, Mancera-Martinez E, Melnikov S, Simonetti A, Myasnikov A, et al. Structural Insights into the Role of Diphthamide on Elongation Factor 2 in mRNA Reading-Frame Maintenance. J Mol Biol. 2018;430(17):2677–87. Epub 2018/06/11. doi: 10.1016/j.jmb.2018.06.006 . 29886014

[27] Hawer H, Utkur K, Arend M, Mayer K, Adrian L, Brinkmann U, et al. Importance of diphthamide modified EF2 for translational accuracy and competitive cell growth in yeast. PLoS One. 2018;13(10):e0205870. Epub 2018/10/20. doi: 10.1371/journal.pone.0205870 ; PubMed Central PMCID: PMC6193676. 30335802 PMC6193676

[28] Nabais Sa MJ, Olson AN, Yoon G, Nimmo GAM, Gomez CM, Willemsen MA, et al. De Novo variants in EEF2 cause a neurodevelopmental disorder with benign external hydrocephalus. Hum Mol Genet. 2021;29(24):3892–9. Epub 2020/12/24. doi: 10.1093/hmg/ddaa270 ; PubMed Central PMCID: PMC7907856. 33355653 PMC7907856

[29] Hekman KE, Yu GY, Brown CD, Zhu H, Du X, Gervin K, et al. A conserved eEF2 coding variant in SCA26 leads to loss of translational fidelity and increased susceptibility to proteostatic insult. Hum Mol Genet. 2012;21(26):5472–83. Epub 2012/09/25. doi: 10.1093/hmg/dds392 ; PubMed Central PMCID: PMC3516132. 23001565 PMC3516132

[30] Ortiz PA, Ulloque R, Kihara GK, Zheng H, Kinzy TG. Translation elongation factor 2 anticodon mimicry domain mutants affect fidelity and diphtheria toxin resistance. J Biol Chem. 2006;281(43):32639–48. doi: 10.1074/jbc.M607076200 . 16950777

[31] Djumagulov M, Demeshkina N, Jenner L, Rozov A, Yusupov M, Yusupova G. Accuracy mechanism of eukaryotic ribosome translocation. Nature. 2021;600(7889):543–6. Epub 20211201. doi: 10.1038/s41586-021-04131-9 ; PubMed Central PMCID: PMC8674143. 34853469 PMC8674143

[32] Milicevic N, Jenner L, Myasnikov A, Yusupov M, Yusupova G. mRNA reading frame maintenance during eukaryotic ribosome translocation. Nature. 2024;625(7994):393–400. Epub 20231129. doi: 10.1038/s41586-023-06780-4 . 38030725

[33] Monkemeyer L, Klaips CL, Balchin D, Korner R, Hartl FU, Bracher A. Chaperone Function of Hgh1 in the Biogenesis of Eukaryotic Elongation Factor 2. Mol Cell. 2019;74(1):88–100 e9. Epub 2019/03/17. doi: 10.1016/j.molcel.2019.01.034 . 30876804

[34] Schopf FH, Huber EM, Dodt C, Lopez A, Biebl MM, Rutz DA, et al. The Co-chaperone Cns1 and the Recruiter Protein Hgh1 Link Hsp90 to Translation Elongation via Chaperoning Elongation Factor 2. Mol Cell. 2019;74(1):73–87 e8. Epub 2019/03/17. doi: 10.1016/j.molcel.2019.02.011 . 30876805

[35] Hohrman K, Goncalves D, Morano KA, Johnson JL. Disrupting progression of the yeast Hsp90 folding pathway at different transition points results in client-specific maturation defects. Genetics. 2021;217(3). Epub 2021/04/01. doi: 10.1093/genetics/iyab009 ; PubMed Central PMCID: PMC8045699. 33789348 PMC8045699

[36] Mercier R, Yama D, LaPointe P, Johnson JL. Hsp90 mutants with distinct defects provide novel insights into cochaperone regulation of the folding cycle. PLoS Genet. 2023;19(5):e1010772. Epub 20230525. doi: 10.1371/journal.pgen.1010772 . 37228112 PMC10246838

[37] Rios EI, Goncalves D, Morano KA, Johnson JL. Quantitative proteomic analysis reveals unique Hsp90 cycle-dependent client interactions. Genetics. 2024;227(2). doi: 10.1093/genetics/iyae057 ; PubMed Central PMCID: PMC11151932. 38606935 PMC11151932

[38] Gietz RD, Sugino A. New yeast-Escherichia coli shuttle vectors constructed with in vitro mutagenized yeast genes lacking six-base pair restriction sites. Gene. 1988;74(2):527–34. doi: 10.1016/0378-1119(88)90185-0 . 3073106

[39] Flom GA, Langner E, Johnson JL. Identification of an Hsp90 mutation that selectively disrupts cAMP/PKA signaling in Saccharomyces cerevisiae. Curr Genet. 2012;58(3):149–63. Epub 2012/03/31. doi: 10.1007/s00294-012-0373-7 . 22461145

[40] Zuehlke AD, Johnson JL. Chaperoning the Chaperone: A Role for the Co-chaperone Cpr7 in Modulating Hsp90 Function in Saccharomyces cerevisiae. Genetics. 2012;191:805–14. Epub 2012/04/17. doi: genetics.112.140319 [pii] doi: 10.1534/genetics.112.140319 . 22505624 PMC3389976

[41] Mattheakis LC, Shen WH, Collier RJ. DPH5, a methyltransferase gene required for diphthamide biosynthesis in Saccharomyces cerevisiae. Mol Cell Biol. 1992;12(9):4026–37. Epub 1992/09/01. doi: 10.1128/mcb.12.9.4026-4037.1992 ; PubMed Central PMCID: PMC360293. 1508200 PMC360293

[42] Christianson TW, Sikorski RS, Dante M, Shero JH, Hieter P. Multifunctional yeast high-copy-number shuttle vectors. Gene. 1992;110(1):119–22. doi: 10.1016/0378-1119(92)90454-w . 1544568

[43] Yan W, Craig EA. The glycine-phenylalanine-rich region determines the specificity of the yeast Hsp40 Sis1. Mol Cell Biol. 1999;19(11):7751–8. PubMed doi: 10.1128/MCB.19.11.7751 . 10523664 PMC84827

[44] Voth WP, Jiang YW, Stillman DJ. New ’marker swap’ plasmids for converting selectable markers on budding yeast gene disruptions and plasmids. Yeast. 2003;20(11):985–93. Epub 2003/08/05. doi: 10.1002/yea.1018 . 12898713

[45] Petropavlovskiy AA, Tauro MG, Lajoie P, Duennwald ML. A Quantitative Imaging-Based Protocol for Yeast Growth and Survival on Agar Plates. STAR Protoc. 2020;1(3):100182. Epub 20201125. doi: 10.1016/j.xpro.2020.100182 ; PubMed Central PMCID: PMC7757406. 33377076 PMC7757406

[46] Tenge VR, Zuehlke AD, Shrestha N, Johnson JL. The Hsp90 cochaperones Cpr6, Cpr7, and Cns1 interact with the intact ribosome. Eukaryot Cell. 2015;14(1):55–63. doi: 10.1128/EC.00170-14 ; PubMed Central PMCID: PMC4279014. 25380751 PMC4279014

[47] Marsh JA, Kalton HM, Gaber RF. Cns1 is an essential protein associated with the hsp90 chaperone complex in Saccharomyces cerevisiae that can restore cyclophilin 40-dependent functions in cpr7Delta cells. Mol Cell Biol. 1998;18(12):7353–9. PubMed doi: 10.1128/MCB.18.12.7353 . 9819422 PMC109317

[48] Dolinski KJ, Cardenas ME, Heitman J. CNS1 encodes an essential p60/Sti1 homolog in Saccharomyces cerevisiae that suppresses cyclophilin 40 mutations and interacts with Hsp90. Mol Cell Biol. 1998;18(12):7344–52. PubMed doi: 10.1128/MCB.18.12.7344 . 9819421 PMC109316

[49] Tesic M, Marsh JA, Cullinan SB, Gaber RF. Functional interactions between Hsp90 and the Co-chaperones Cns1 and Cpr7 in saccharomyces cerevisiae. J Biol Chem. 2003;278(35):32692–701. PubMed doi: 10.1074/jbc.M304315200 . 12788914

[50] Mattheakis LC, Sor F, Collier RJ. Diphthamide synthesis in Saccharomyces cerevisiae: structure of the DPH2 gene. Gene. 1993;132(1):149–54. Epub 1993/09/30. doi: 10.1016/0378-1119(93)90528-b . 8406038

[51] Meyer P, Prodromou C, Hu B, Vaughan C, Roe SM, Panaretou B, et al. Structural and functional analysis of the middle segment of hsp90. Implications for ATP hydrolysis and client protein and cochaperone interactions. Mol Cell. 2003;11(3):647–58. PubMed doi: 10.1016/s1097-2765(03)00065-0 . 12667448

[52] Ali MM, Roe SM, Vaughan CK, Meyer P, Panaretou B, Piper PW, et al. Crystal structure of an Hsp90-nucleotide-p23/Sba1 closed chaperone complex. Nature. 2006;440(7087):1013–7. PubMed doi: 10.1038/nature04716 . 16625188 PMC5703407

[53] Mercier R, Wolmarans A, Schubert J, Neuweiler H, Johnson JL, LaPointe P. The conserved NxNNWHW motif in Aha-type co-chaperones modulates the kinetics of Hsp90 ATPase stimulation. Nat Commun. 2019;10(1):1273. Epub 2019/03/22. doi: 10.1038/s41467-019-09299-3 ; PubMed Central PMCID: PMC6426937. 30894538 PMC6426937

[54] Johnson JL, Halas A, Flom G. Nucleotide-Dependent Interaction of Saccharomyces cerevisiae Hsp90 with the Cochaperone Proteins Sti1, Cpr6, and Sba1. Mol Cell Biol. 2007;27(2):768–76. PubMed doi: 10.1128/MCB.01034-06 . 17101799 PMC1800796

[55] Ortiz PA, Kinzy TG. Dominant-negative mutant phenotypes and the regulation of translation elongation factor 2 levels in yeast. Nucleic Acids Res. 2005;33(18):5740–8. doi: 10.1093/nar/gki882 ; PubMed Central PMCID: PMC1253829. 16214807 PMC1253829

[56] Fenwick MK, Dong M, Lin H, Ealick SE. The Crystal Structure of Dph2 in Complex with Elongation Factor 2 Reveals the Structural Basis for the First Step of Diphthamide Biosynthesis. Biochemistry. 2019;58(43):4343–51. Epub 2019/10/01. doi: 10.1021/acs.biochem.9b00718 ; PubMed Central PMCID: PMC7857147. 31566354 PMC7857147

[57] Sahi C, Craig EA. Network of general and specialty J protein chaperones of the yeast cytosol. Proc Natl Acad Sci U S A. 2007;104(17):7163–8. Epub 2007/04/18. doi: 0702357104 [pii] doi: 10.1073/pnas.0702357104 ; PubMed Central PMCID: PMC1855418. 17438278 PMC1855418

[58] Uthman S, Bar C, Scheidt V, Liu S, ten Have S, Giorgini F, et al. The amidation step of diphthamide biosynthesis in yeast requires DPH6, a gene identified through mining the DPH1-DPH5 interaction network. PLoS Genet. 2013;9(2):e1003334. Epub 2013/03/08. doi: 10.1371/journal.pgen.1003334 ; PubMed Central PMCID: PMC3585130. 23468660 PMC3585130

[59] Workman P, Clarke PA, Al-Lazikani B. Blocking the survival of the nastiest by HSP90 inhibition. Oncotarget. 2016;7(4):3658–61. doi: 10.18632/oncotarget.6971 ; PubMed Central PMCID: PMC4826159. 26820296 PMC4826159

[60] Whitesell L, Lindquist SL. HSP90 and the chaperoning of cancer. Nat Rev Cancer. 2005;5(10):761–72. PubMed doi: 10.1038/nrc1716 . 16175177

[61] Cowen LE, Singh SD, Kohler JR, Collins C, Zaas AK, Schell WA, et al. Harnessing Hsp90 function as a powerful, broadly effective therapeutic strategy for fungal infectious disease. Proc Natl Acad Sci U S A. 2009;106(8):2818–23. PubMed doi: 10.1073/pnas.0813394106 . 19196973 PMC2650349

[62] Butler LM, Ferraldeschi R, Armstrong HK, Centenera MM, Workman P. Maximizing the Therapeutic Potential of HSP90 Inhibitors. Molecular cancer research: MCR. 2015;13(11):1445–51. doi: 10.1158/1541-7786.MCR-15-0234 ; PubMed Central PMCID: PMC4645455. 26219697 PMC4645455

[63] Trepel J, Mollapour M, Giaccone G, Neckers L. Targeting the dynamic HSP90 complex in cancer. Nat Rev Cancer. 2010;10(8):537–49. Epub 2010/07/24. doi: nrc2887 [pii] doi: 10.1038/nrc2887 . 20651736 PMC6778733

[64] Schaffrath R, Abdel-Fattah W, Klassen R, Stark MJ. The diphthamide modification pathway from Saccharomyces cerevisiae—revisited. Mol Microbiol. 2014;94(6):1213–26. Epub 2014/10/30. doi: 10.1111/mmi.12845 . 25352115

[65] Webb TR, Cross SH, McKie L, Edgar R, Vizor L, Harrison J, et al. Diphthamide modification of eEF2 requires a J-domain protein and is essential for normal development. J Cell Sci. 2008;121(Pt 19):3140–5. Epub 2008/09/04. doi: 10.1242/jcs.035550 ; PubMed Central PMCID: PMC2592597. 18765564 PMC2592597

[66] Youker RT, Walsh P, Beilharz T, Lithgow T, Brodsky JL. Distinct roles for the Hsp40 and Hsp90 molecular chaperones during cystic fibrosis transmembrane conductance regulator degradation in yeast. Mol Biol Cell. 2004;15(11):4787–97. doi: 10.1091/mbc.e04-07-0584 ; PubMed Central PMCID: PMC524727. 15342786 PMC524727

[67] Kimura Y, Yahara I, Lindquist S. Role of the protein chaperone YDJ1 in establishing Hsp90-mediated signal transduction pathways. Science. 1995;268(5215):1362–5. PubMed doi: 10.1126/science.7761857 . 7761857

[68] Kampinga HH, Craig EA. The HSP70 chaperone machinery: J proteins as drivers of functional specificity. Nat Rev Mol Cell Biol. 2010;11(8):579–92. doi: 10.1038/nrm2941 ; PubMed Central PMCID: PMC3003299. 20651708 PMC3003299

[69] Zierer BK, Rubbelke M, Tippel F, Madl T, Schopf FH, Rutz DA, et al. Importance of cycle timing for the function of the molecular chaperone Hsp90. Nat Struct Mol Biol. 2016;23(11):1020–8. doi: 10.1038/nsmb.3305 . 27723736 PMC6248305

[70] Mishra P, Flynn JM, Starr TN, Bolon DN. Systematic Mutant Analyses Elucidate General and Client-Specific Aspects of Hsp90 Function. Cell reports. 2016;15(3):588–98. doi: 10.1016/j.celrep.2016.03.046 ; PubMed Central PMCID: PMC4838542. 27068472 PMC4838542

[71] Nathan DF, Vos MH, Lindquist S. Identification of SSF1, CNS1, and HCH1 as multicopy suppressors of a Saccharomyces cerevisiae Hsp90 loss-of-function mutation. Proc Natl Acad Sci U S A. 1999;96(4):1409–14. PubMed doi: 10.1073/pnas.96.4.1409 . 9990037 PMC15476

[72] Chang HC, Nathan DF, Lindquist S. In vivo analysis of the Hsp90 cochaperone Sti1 (p60). Mol Cell Biol. 1997;17(1):318–25. PubMed doi: 10.1128/MCB.17.1.318 . 8972212 PMC231756

[73] Flom G, Weekes J, Williams JJ, Johnson JL. Effect of mutation of the tetratricopeptide repeat and aspartate-proline 2 domains of Sti1 on Hsp90 signaling and interaction in Saccharomyces cerevisiae. Genetics. 2006;172(1):41–51. PubMed doi: 10.1534/genetics.105.045815 . 16219779 PMC1456168

[74] Tenge VR, Knowles J, Johnson JL. The ribosomal biogenesis protein Utp21 interacts with Hsp90 and has differing requirements for Hsp90-associated proteins. PLoS One. 2014;9(3):e92569. doi: 10.1371/journal.pone.0092569 ; PubMed Central PMCID: PMC3960262. 24647762 PMC3960262

[75] Albanese V, Yam AY, Baughman J, Parnot C, Frydman J. Systems analyses reveal two chaperone networks with distinct functions in eukaryotic cells. Cell. 2006;124(1):75–88. PubMed doi: 10.1016/j.cell.2005.11.039 . 16413483

[76] Wade CH, Umbarger MA, McAlear MA. The budding yeast rRNA and ribosome biosynthesis (RRB) regulon contains over 200 genes. Yeast. 2006;23(4):293–306. Epub 2006/03/18. doi: 10.1002/yea.1353 . 16544271

[77] Duina AA, Kalton HM, Gaber RF. Requirement for Hsp90 and a CyP-40-type cyclophilin in negative regulation of the heat shock response. J Biol Chem. 1998;273(30):18974–8. PubMed doi: 10.1074/jbc.273.30.18974 . 9668076

[78] Schuster M, Schnell L, Feigl P, Birkhofer C, Mohr K, Roeder M, et al. The Hsp90 machinery facilitates the transport of diphtheria toxin into human cells. Scientific reports. 2017;7(1):613. Epub 2017/04/06. doi: 10.1038/s41598-017-00780-x ; PubMed Central PMCID: PMC5429619. 28377614 PMC5429619

[79] Flom G, Weekes J, Johnson JL. Novel interaction of the Hsp90 chaperone machine with Ssl2, an essential DNA helicase in Saccharomyces cerevisiae. Curr Genet. 2005;47(6):368–80. PubMed doi: 10.1007/s00294-005-0580-6 . 15871019 PMC2267864

