# Supplementary material for: Hsp90 and cochaperones have two genetically distinct roles in regulating eEF2 function
Source: PLoS Genet. 2024 Dec 9;20(12):e1011508. doi: 10.1371/journal.pgen.1011508 (PMC11651573; doi:10.1371/journal.pgen.1011508)
Supplement: S1 Table — (DOCX) [file pgen.1011508.s001.docx]

**Supporting Information**

**S1 Table. Strain list**

| Strain name | Relevant genotype | Reference |
| --- | --- | --- |
| JJ762 (wild-type) | *trp1-1  ura3-1  leu2-3,112 his3-11,15 ade2-1   met2-Δ1 lys2-Δ2* | [1] |
| JJ21 | *cns1::TRP1 /URA3-CNS1* | [2] |
| JJ1115 | *cpr7::kanR* | [2] |
| JJ1465 | *hgh1:kanR* | This study |
| JJ1449 | *dph2::kanR* | This study |
| JJ623 | *sti1::MET2* | [3] |
| JJ73 | *aha1::kanR* | This study |
| JJ543 | *sba1::kanR* | [4] |
| JJ1138 | *cpr6::kanR* | [2] |
| JJ816 | *hsc82::LEU2 hsp82::LEU2/URA-HSP82* | [5] |
| JJ117 | *hsc82::kanR hsp82::kanR/URA-HSP82* | This study |
| JJ1471 | *hgh1::kanR hsc82::LEU2 hsp82::LEU2/URA-HSP82* | This study |
| JJ1472 | *eft1::kanR eft2::kanR/URA-EFT1* | This study |
| JJ1481 | *hgh1::TRP1 eft1::kanR eft2::kanR/URA-EFT1.* | This study |
| Y1900 | *jjj3::LEU2* | [6] |

1. Yan W, Craig EA. The glycine-phenylalanine-rich region determines the specificity of the yeast Hsp40 Sis1. Mol Cell Biol. 1999;19(11):7751-8. PubMed PMID: 10523664.

2. Tenge VR, Zuehlke AD, Shrestha N, Johnson JL. The Hsp90 cochaperones Cpr6, Cpr7, and Cns1 interact with the intact ribosome. Eukaryot Cell. 2015;14(1):55-63. doi: 10.1128/EC.00170-14. PubMed PMID: 25380751; PubMed Central PMCID: PMC4279014.

3. Flom G, Weekes J, Williams JJ, Johnson JL. Effect of mutation of the tetratricopeptide repeat and aspartate-proline 2 domains of Sti1 on Hsp90 signaling and interaction in Saccharomyces cerevisiae. Genetics. 2006;172(1):41-51. PubMed PMID: 16219779.

4. Noddings CM, Wang RY, Johnson JL, Agard DA. Structure of Hsp90-p23-GR reveals the Hsp90 client-remodelling mechanism. Nature. 2022;601(7893):465-9. Epub 2021/12/24. doi: 10.1038/s41586-021-04236-1. PubMed PMID: 34937936; PubMed Central PMCID: PMCPMC8994517.

5. Johnson JL, Halas A, Flom G. Nucleotide-Dependent Interaction of Saccharomyces cerevisiae Hsp90 with the Cochaperone Proteins Sti1, Cpr6, and Sba1. Mol Cell Biol. 2007;27(2):768-76. PubMed PMID: 17101799.

6. Sahi C, Craig EA. Network of general and specialty J protein chaperones of the yeast cytosol. Proc Natl Acad Sci U S A. 2007;104(17):7163-8. Epub 2007/04/18. doi: 0702357104 [pii]

10.1073/pnas.0702357104. PubMed PMID: 17438278; PubMed Central PMCID: PMC1855418.
