# Supplementary material for: Hsp90 and cochaperones have two genetically distinct roles in regulating eEF2 function
Source: PLoS Genet. 2024 Dec 9;20(12):e1011508. doi: 10.1371/journal.pgen.1011508 (PMC11651573; doi:10.1371/journal.pgen.1011508)
Supplement: S2 Table — General classification of groups with specific defects listed. Shaded columns represent new data from this study. (DOCX) [file pgen.1011508.s002.docx]

**S2 Table. Information about Hsc82 mutants used in this study**. General classification of groups with specific defects listed. Shaded columns represent new data from this study.

| **Hsc82 mutation** | **Group** | **Effect on ability of Hsc82 to form transitional complexes** [1] | **Effect of *HCH1* overexpression**  **on growth** [2] | **Effect on eEF2 steady state level** (this study) | **Effect on sensitivity to DT** (this study) |
| --- | --- | --- | --- | --- | --- |
| R46G | Loading | Reduced interaction with Hsp70. | Sharp decrease at 30°C | nd | reduced |
| G309S | Loading |  | sharp decrease at 30°C | decreased | reduced |
|  | | | | | |
| S481Y | Closing | Reduced interaction with Sba1 and Cpr6. | Sharp decrease at 30°C | nd | nd |
| A583T | Closing |  | sharp decrease at 30°C | decreased | reduced |
|  | | | | | |
| S25P | Reopening | Normal interactions with Hsp70, Sba1, and Cpr6. In regions implicated in regulation of ATP hydrolysis. | Rescued 37°C defect | strong decrease | none |
| K102E | Reopening |  | rescued 37°C defect | strong decrease | none |
| E377A | Reopening |  | rescued 37°C defect | strong decrease | nd |
| L379S | Reopening |  | rescued 37°C defect | strong decrease | nd |
| Q380K | Reopening |  | rescued 37°C defect | strong decrease | nd |
|  | | | | | |
| G424D | Other | Altered interactions with Sti1 and Hsp70. |  | None | none |

*nd= not determined

1. Hohrman K, Goncalves D, Morano KA, Johnson JL. Disrupting progression of the yeast Hsp90 folding pathway at different transition points results in client-specific maturation defects. Genetics. 2021;217(3). Epub 2021/04/01. doi: 10.1093/genetics/iyab009. PubMed PMID: 33789348; PubMed Central PMCID: PMCPMC8045699.

2. Mercier R, Yama D, LaPointe P, Johnson JL. Hsp90 mutants with distinct defects provide novel insights into cochaperone regulation of the folding cycle. PLoS Genet. 2023;19(5):e1010772. Epub 20230525. doi: 10.1371/journal.pgen.1010772. PubMed PMID: 37228112.
