# Supplementary figures and images for: Hsp90 and cochaperones have two genetically distinct roles in regulating eEF2 function
Source: PLoS Genet. 2024 Dec 9;20(12):e1011508. doi: 10.1371/journal.pgen.1011508 (PMC11651573; doi:10.1371/journal.pgen.1011508)

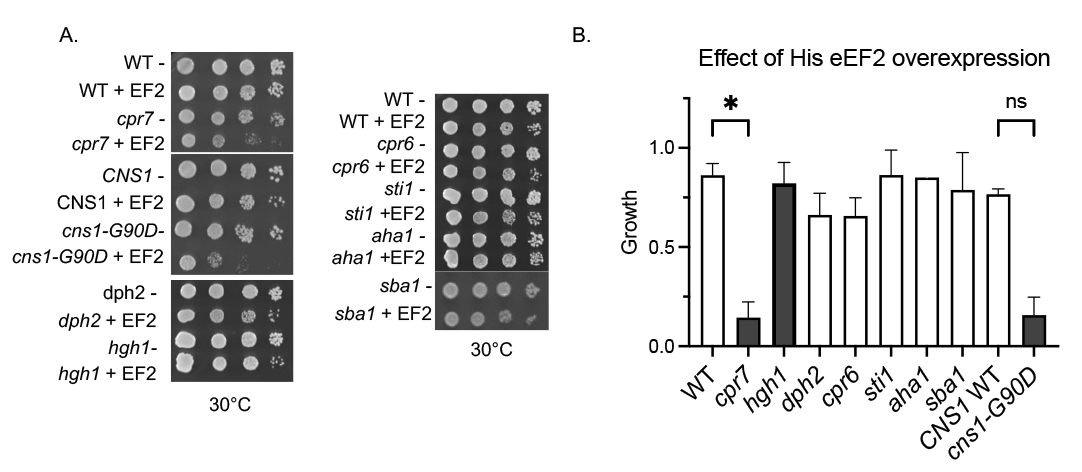

Supplement: S1 Fig — A. A plasmid expressing wild-type His-eEF2 or empty vector was transformed into the strains shown as in Fig 1. Transformants were grown overnight in selective media, serially diluted 10-fold and grown on selective media for 2 days at 30°C. B. Growth of biological replicates expressing eEF2 normalized to cells expressing empty vector, with representative pictures of each shown. Cochaperones previously shown to be required for eEF2 folding, are shaded in dark gray. Statistical significance was evaluated with GraphPad Prism using Mixed-effects analysis (* P ≤ 0.05). Non-significant values (P ≥ 0.05 not shown). (TIF) [file pgen.1011508.s003.tif]

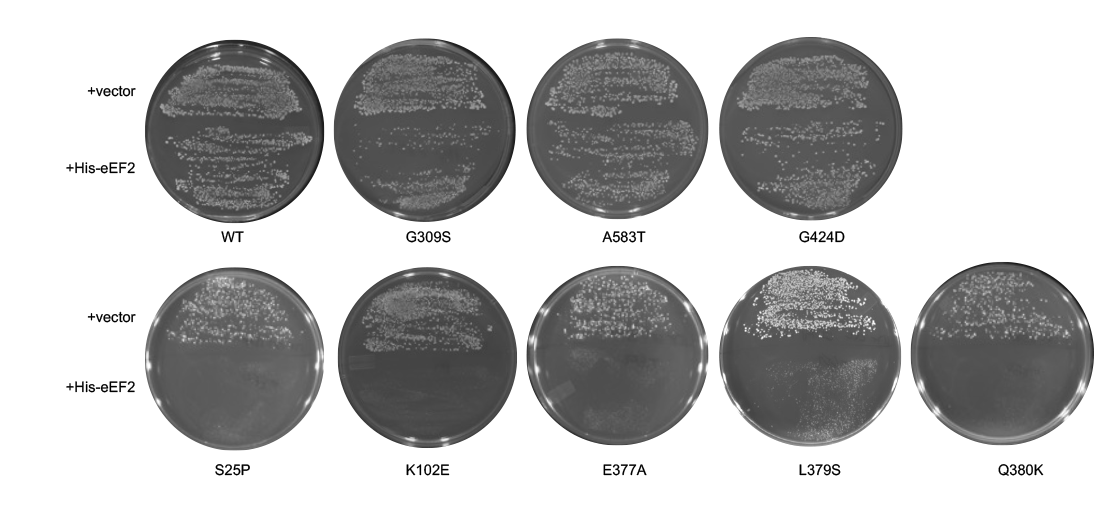

Supplement: S2 Fig — A plasmid expressing wild-type His-eEF2 (bottom) or empty vector (top) was transformed into hsc82hsp82 strains expressing either wild-type Hsc82 or the indicated mutant. Pictures were taken after growth on selective media for 2 or 3 days at 30°C. (TIF) [file pgen.1011508.s004.tif]

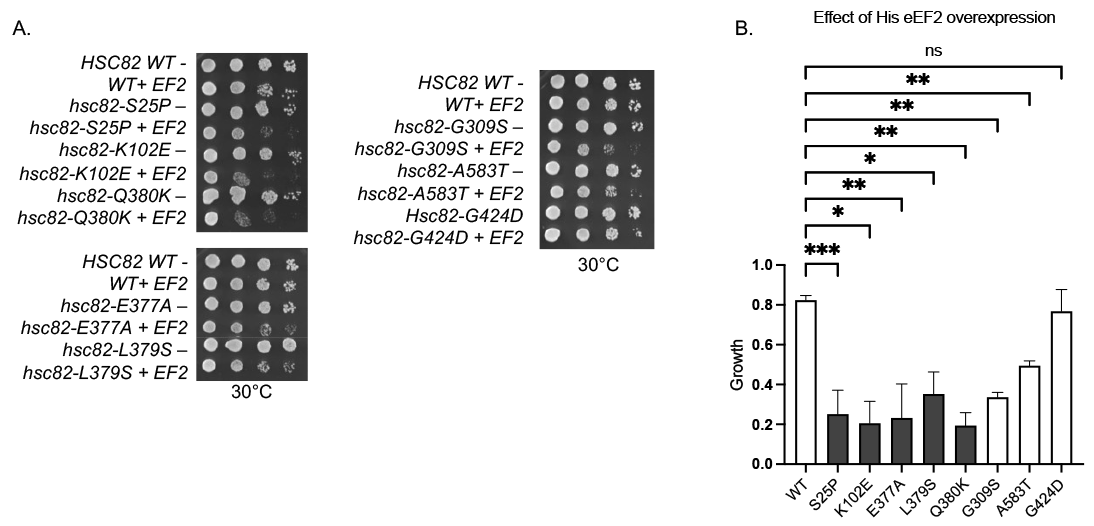

Supplement: S3 Fig — A plasmid expressing wild-type His-eEF2 or empty vector was transformed into the strains as in S2 Fig. Transformants were grown overnight in selective media, serially diluted 10-fold and grown on selective media for 2 days at 30°C. Growth of biological replicates expressing EF2 was normalized to cells expressing empty vector, with representative pictures of each shown. Hsc82 mutants in the reopening category are shaded in dark gray. Statistical significance was evaluated with GraphPad Prism using Mixed-effects analysis (* P ≤ 0.05; ** P ≤ 0.01; *** P ≤ 0.001). Non-significant values (P ≥ 0.05 not shown). (TIF) [file pgen.1011508.s005.tif]

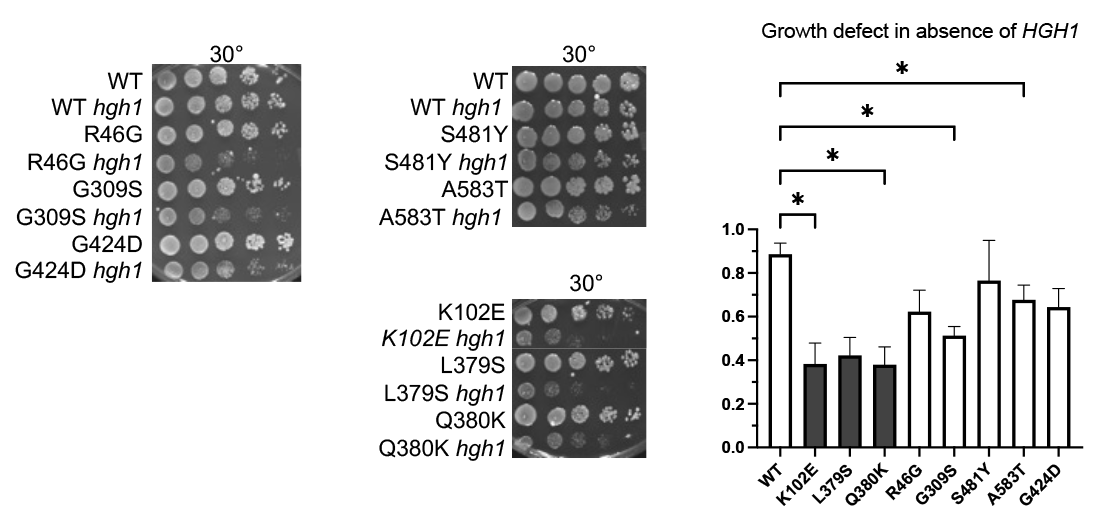

Supplement: S4 Fig — Colonies were picked from the 5-FOA plates in Fig 2, grown overnight in rich media, serially diluted 10-fold and grown on rich media (YPD) for 2 days at 30°C. Growth of hgh1hsc82 hsp82 strains expressing each hsc82 mutant was normalized to the growth of the same mutant in an hsc82hsp82 strain. Three biological replicates were obtained, with representative pictures of each shown. Hsc82 mutants in the reopening category are shaded in dark gray. Statistical significance was evaluated with GraphPad Prism using Mixed-effects analysis (* P ≤ 0.05). Non-significant values (P ≥ 0.05 not shown). (TIF) [file pgen.1011508.s006.tif]

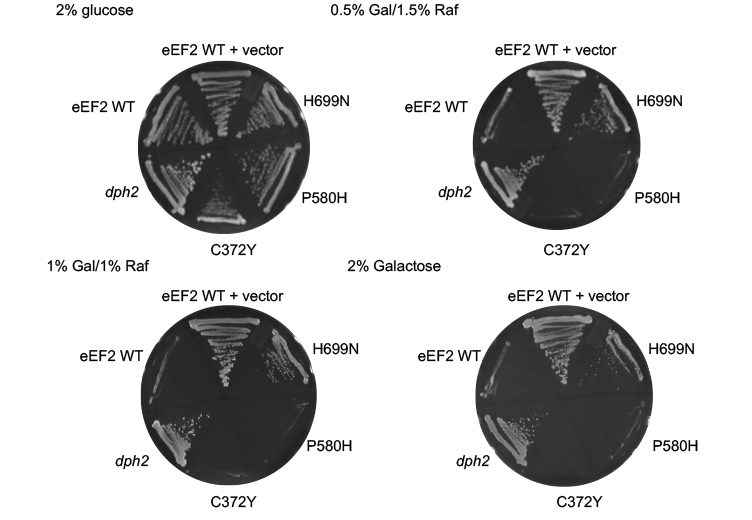

Supplement: S5 Fig — Strain 1472 (eft1eft2) expressing wild-type or mutant His-EF2 was transformed with plasmid pLMY101. Strains were grown on selective (- uracil) plates containing 0.5%, 1%, or 2% galactose or 2% glucose (as a control) and grown for four days at 30°C. As needed, raffinose was added so that the total of raffinose plus galactose was 2%. Three biological replicates were obtained, with representative pictures of each shown. (TIF) [file pgen.1011508.s007.tif]
